# Reconstitution of mammalian mitochondrial translation system capable of correct initiation and long polypeptide synthesis from leaderless mRNA

**DOI:** 10.1093/nar/gkaa1165

**Published:** 2020-12-09

**Authors:** Muhoon Lee, Noriko Matsunaga, Shiori Akabane, Ippei Yasuda, Takuya Ueda, Nono Takeuchi-Tomita

**Affiliations:** Department of Computational Biology and Medical Sciences, Graduate School of Frontier Sciences, The University of Tokyo, 5-1-5, Kashiwanoha, Kashiwa-shi, Chiba 277-8562, Japan; Department of Computational Biology and Medical Sciences, Graduate School of Frontier Sciences, The University of Tokyo, 5-1-5, Kashiwanoha, Kashiwa-shi, Chiba 277-8562, Japan; Department of Computational Biology and Medical Sciences, Graduate School of Frontier Sciences, The University of Tokyo, 5-1-5, Kashiwanoha, Kashiwa-shi, Chiba 277-8562, Japan; Department of Life Science, Rikkyo University, Tokyo, 171-8501, Japan; Department of Computational Biology and Medical Sciences, Graduate School of Frontier Sciences, The University of Tokyo, 5-1-5, Kashiwanoha, Kashiwa-shi, Chiba 277-8562, Japan; Department of Computational Biology and Medical Sciences, Graduate School of Frontier Sciences, The University of Tokyo, 5-1-5, Kashiwanoha, Kashiwa-shi, Chiba 277-8562, Japan; Department of Integrative Bioscience and Biomedical Engineering, Graduate School of Science and Engineering, Waseda University, Tokyo, Shinjuku 162-8480, Japan; Department of Computational Biology and Medical Sciences, Graduate School of Frontier Sciences, The University of Tokyo, 5-1-5, Kashiwanoha, Kashiwa-shi, Chiba 277-8562, Japan

## Abstract

Mammalian mitochondria have their own dedicated protein synthesis system, which produces 13 essential subunits of the oxidative phosphorylation complexes. We have reconstituted an *in vitro* translation system from mammalian mitochondria, utilizing purified recombinant mitochondrial translation factors, 55S ribosomes from pig liver mitochondria, and a tRNA mixture from either *Escherichia coli* or yeast. The system is capable of translating leaderless mRNAs encoding model proteins (DHFR and nanoLuciferase) or some mtDNA-encoded proteins. We show that a leaderless mRNA, encoding nanoLuciferase, is faithfully initiated without the need for any auxiliary factors other than IF-2mt and IF-3mt. We found that the ribosome-dependent GTPase activities of both the translocase EF-G1mt and the recycling factor EF-G2mt are insensitive to fusidic acid (FA), the translation inhibitor that targets bacterial EF-G homologs, and consequently the system is resistant to FA. Moreover, we demonstrate that a polyproline sequence in the protein causes 55S mitochondrial ribosome stalling, yielding ribosome nascent chain complexes. Analyses of the effects of the Mg concentration on the polyproline-mediated ribosome stalling suggested the unique regulation of peptide elongation by the mitoribosome. This system will be useful for analyzing the mechanism of translation initiation, and the interactions between the nascent peptide chain and the mitochondrial ribosome.

## INTRODUCTION

Mitochondria produce most of the energy used by mammalian cells through oxidative phosphorylation. The oxidative phosphorylation (OXPHOS) system is composed of five multi-subunit complexes located within the inner mitochondrial membrane, and comprises nuclear- and mitochondrial DNA (mtDNA)-encoded polypeptides. The mitochondrially encoded polypeptides include seven subunits of Complex I (NADH:ubiquinone oxidoreductase), one subunit of Complex III (coenzyme Q-cytochrome *c* reductase), three subunits of Complex IV (cytochrome *c* oxidase), and two subunits of Complex V (ATP synthase). These 13 membrane subunits are synthesized by the mitochondrial protein synthesis machineries and function in complexes with the nuclear-encoded polypeptides, which are synthesized in the cellular cytosol and subsequently imported into mitochondria (1). The coordinated regulation of mitochondrial and cytoplasmic protein synthesis is required for the proper function of the OXPHOS system. Defects in mitochondrial protein synthesis often lead to various human diseases associated with deficiencies in oxidative phosphorylation (2–4). Some side-effects of drugs are caused by drug mis-targeting to mitochondrial protein synthesis machineries, and the inhibition of mitochondrial protein synthesis (5,6).

The mammalian mitochondrial protein synthesis system utilizes a distinct set of ribosomes with low sedimentation coefficients of 55S, and consist of 28S small subunit and 39S large subunit (7). Mammalian mitochondrial ribosomes (mitoribosomes) have a reduced rRNA content (25–30%RNA) (8), and contain three rRNA species; 12S in the small subunit, 16S and mt-tRNA(Val) in the large subunit (9,10). Mitoribosomes are relatively protein-rich, and the additional and/or larger proteins of the mitoribosome are thought to compensate for the function of the smaller rRNAs (11–18). While the architectures of the catalytic cores of the peptidyl transferase and decoding centers are conserved, there are a number of unique structural features in mitoribosomes, including the absence of anti-SD sequences and different components forming the mRNA-entrance gate (9,19–24). One remarkable feature is the architecture of the polypeptide exit tunnel (PET), which may have evolved to facilitate the co-translational membrane insertion of nascent polypeptides. Notably, in the yeast mitoribosome, nascent polypeptides emerge from a site ∼3.5 nm away from the canonical exit site, and the canonical PET is blocked by the long mitoribosome-specific extension of mL23 (25). In mammalian mitoribosome, the polypeptide exit site observed in the yeast mitoribosome is blocked by the 16S rRNA. The putative polypeptide accessible site (PAS), a lateral opening 25 Å away from the canonical exit site, is also partially blocked by mitoribosome-specific protein components (21,24). Even more intriguing is the fact that mL45, the counterpart of yeast Mba1, a membrane-bound mitoribosome receptor, inserts its tail into the canonical PET and completely blocks the tunnel all the way up to the peptidyl transferase center (26). Taken together, the precise pathway by which the nascent polypeptide is extruded from the mitoribosome to the membrane-insertion machinery remains enigmatic.

Since mitochondria originated from endosymbiotic ancient bacteria, the mechanism of protein synthesis in mitochondria, in principle, resembles that in bacteria. However, as explained below, previous researches have also revealed a number of differences. So far, the individual processes of the mammalian mitochondrial protein synthesis system - initiation, elongation, termination and ribosome recycling - have been reconstituted *in vitro* using mitoribosomes and translation factors. Initiation complex formation has been investigated by analyzing fMet-tRNA binding to mitoribosomes in the presence of short mRNA fragments (27–29). Two mitochondrial translation initiation factors have been identified, IF-2mt and IF-3mt. In the current model (29), IF-3mt initially binds to the 55S mitoribosome, and dissociates it into the subunits forming the [IF-3mt:28S] complex; mitochondrial Pre-Initiation Complex 1 (mtPIC-1). IF-3mt induces the structural rearrangement in the 28S subunit, facilitating the IF-2mt binding to mtPIC-1, that results in the [IF-2mt:IF-3mt:28S] complex; mitochondrial Pre-Initiation Complex 2 (mtPIC-2). Since the binding of IF-3mt and fMet-tRNA on the 28S subunit is mutually exclusive, IF-3mt release from mtPIC-2 then allows the assembly of mRNA, fMet-tRNA and 39S subunit. Consequently, the [55S:fMet-tRNA:mRNA] complex, the complete initiation complex, is formed and proceeds to the elongation process. Here, the exact timing of mRNA- and fMet-tRNA-binding to the 28S subunit, whether it precedes or follows the 39S subunit binding, is still unknown. Thus, whether mitochondrial mRNA is initiated with 28S subunit or with 55S monosome is not known. It should be emphasized that IF-3mt has a ribosome dissociation activity in addition to the anti-association activity of bacterial IF-3, and is thought to actively provide 28S small subunits (30). Mitochondria lack the homolog of bacterial IF-1, a highly conserved essential initiation factor, but the role of this factor is replaced by an insertion found in IF-2mt (26,31,32). Most mammalian mitochondrial mRNAs are leaderless, and thus lack the 5′ leader sequence to promote mRNA recruitment to the ribosome (33). While mammalian mitoribosomes reportedly select the 5′ terminal AUG codon on leaderless mRNA preferentially (28), the question still remains as to whether additional factors, such as transcript-specific translation activators, are required to ensure the translation initiation fidelity of leaderless mRNA in mammalian mitochondria.

The peptide elongation process has primarily been studied by measuring the ribosome binding of Phe-tRNA in the presence of poly(U) mRNA and the poly(U)-dependent poly(Phe) synthesis (34). The aminoacyl-tRNA is delivered to the ribosomal A-site by EF-Tumt. Following the peptide-transfer reaction, translocation is catalyzed by EF-G1mt. EF-G1mt is an exclusive translocase, in contrast to the bacterial EF-G, which is responsible for both translocation and ribosome recycling (35). EF-Tsmt functions as a GDP:GTP exchange factor for EF-Tumt. A modified poly(U)-dependent poly(Phe) synthesis system has been applied to study the function of mt-tRNA^Ser^ (AGY) with an unusual structure lacking the entire D-loop (36). The function of mitochondria-specific modification of 5-formylcytidine (f^5^C) in mt-tRNA^Met^ was investigated by oligo(AUN)-dependent oligo(Met) synthesis system (37).

During the termination and ribosome recycling process, RF-1Lmt/mtRF1a recognizes the stop codon (UAA or UAG) and promotes the peptide release reaction. The ribosomes are subsequently dissociated into subunits for the next round of translation by the concerted actions of RRFmt and EF-G2mt. EF-G2mt, a second mitochondrial homolog of bacterial EF-G, is an exclusive recycling factor that lacks translocation activity (35). The peptide release activity of RF-1Lmt/mtRF1a has been detected by analyzing the ribosome-dependent peptidyl-tRNA hydrolysis activity (38–40). The activities of EF-G2mt and RRFmt have been investigated by examining the 55S dissociation reaction and their ability to promote multi-round translation (35). There is no homolog of bacterial RF-3, and the mechanism by which RF1Lmt/mtRF1a exits the ribosome following the peptide release reaction is unclear. Besides RF-1Lmt/mtRF1a, there are four RF-family proteins in mammalian mitochondria: mtRF1, ICT1, C12orf65 and mS35 (41), but their precise functions remain to be clarified.

In the present study, we have reconstituted an *in vitro* translation system from mammalian mitochondria, utilizing the purified mitochondrial translation factors (IF-2mt, IF-3mt, EF-Tumt, EF-Tsmt, EF-G1mt, EF-G2mt, RF-1Lmt/mtRF1a and RRFmt) and 55S mitoribosomes, whereby correct initiation can proceed and translation of a defined ORF is allowed from leaderless mRNA. The developed system is capable of synthesizing proteins long enough to pass through the peptide tunnel, and the lengths and sequences of the nascent peptide chains can be controlled. Ribosome nascent chain complexes (RNC) can be obtained by utilizing the polyproline-mediated ribosome stalling. The system facilitates the biochemical analyses of translation initiation in mammalian mitochondria, and the regulation of peptide elongation through the interaction between the nascent peptide chain and the peptide tunnel of the mitoribosome. The system also provides a framework for studying the functions of mitochondrial tRNAs, and the co-translational membrane insertion of proteins in mammalian mitochondria.

## MATERIALS AND METHODS

### Preparation of template DNA and mRNA for translation

The DNA templates used for the translation were chemically synthesized (Thermo Fisher Scientific), and the mRNAs were transcribed using T7 RNA polymerase. See Supplementary Tables S1 and S2 for the sequences of the templates.

### Purification of components used in the reconstituted mammalian mitochondrial translation system

55S ribosomes were prepared from pig liver mitochondria (42), with slight modification; crude mitoribosomes (70 A_260_ units in 600 μl) were treated with 0.5 mM (final concentration) puromycin at 27°C for 15 min, just before loading onto the sucrose density gradient for 55S purification. The *Escherichia coli* tRNA mixture was purchased from Roche. The yeast tRNA mixture and aminoacyl-tRNAs were prepared as described (43). For the other components, see Supplementary Table S3.

### Reconstitution of the mammalian mitochondrial translation system

Unless otherwise specified, the standard translation mixtures (10 μl) contained 50 mM HEPES-KOH [pH 7.5], 100 mM potassium glutamate, 7 mM Mg(OAc)_2_, 2 mM spermidine, 0.1 mM spermine, 1 mM DTT, 0.15 mM of each amino acid (except methionine and cysteine), 0.05 mM methionine, 0.1 mM cysteine, 1 mM ATP, 1 mM GTP, 20 mM creatine phosphate, 0.1 μg 10-formyl-5,6,7,8-tetrahydrofolic acid, 100 nM creatine kinase, 20 nM myokinase, 15 nM nucleoside–diphosphate kinase, 15 nM pyrophosphatase, 0.5 μM IF-2mt, 0.5 μM IF-3mt, 5 μM EF-Tumt, 1 μM EF-Tsmt, 0.5 μM EF-G1mt, 0.5 μM EF-G2mt, 0.5 μM RF-1Lmt, 0.5 μM RRFmt, 5 μM methionyl-tRNA transformylase (*E. coli* MTF), 0.2 μM 55S ribosome, 0.045 A_260_ units yeast aminoacyl-tRNA mixture, and 0.1 μM mRNA. Where indicated, 0.5 μL of [^35^S]methionine (>37 TBq/mmol, 400 MBq/mL) was included, instead of unlabeled methionine. The reaction mixture was incubated at 37°C for the indicated period of time. After the samples were treated with RNase A (final concentration 0.2 mg/ml) at 30°C for 10 min, the samples were resolved by Tricine SDS-PAGE. The RNase A treatment was omitted for peptide release assays (Supplementary Figures S3 and S6). We note that yeast initiator methionyl-tRNA, and its formylated form fMet-tRNA, are good substrates for *E. coli* MTF and IF-2mt, respectively (44,45).

To couple aminoacylation and/or transcription with the translation reaction, we modified the reaction mixture. The reaction contained 13 mM Mg(OAc)_2_, 2 mM spermidine, 0.1 mM spermine, 2 mM ATP, 2 mM GTP, 1 mM CTP and 1 mM UTP. When aminoacylation was coupled, 30–300 units of each *E. coli* ARS (46) and 0.56 A_260_ units *E. coli* tRNA mixture were included, instead of the yeast aminoacyl-tRNA mixture. For the transcription-translation coupled reaction, 30 nM T7 RNA polymerase and 10 nM template DNA were included, instead of the mRNA.

To reduce manipulation errors, combine as many components as possible and add in one step. For example, a reaction mixture for multiple samples is prepared and aliquoted into mRNA previously prepared in different tubes.

The sensitivity of the reconstituted mitochondrial translation system for the antibiotics (fusidic acid, chloramphenicol and cycloheximide) has been examined to exclude the possibility of contaminating cytosolic ribosomes (Supplementary Figure S1).

### nanoLuciferase assay

The enzymatic activity of the products was assessed with a Nano-Glo Luciferase Assay System (Promega). The 2 μl reaction was stopped by adding 18 μl of stop solution (20 mM HEPES–KOH [pH 7.5], 100 mM KOAc, 2 mM Mg(OAc)_2_, 0.1 mg/ml RNase A), and then mixed with 20 μl of substrate in a white 96-well half area plate. After an incubation for 17 min at room temperature, the luminescence was detected with a GloMax Multi Detection System (Promega).

### Measurement of GTPase activity

Ribosome-dependent GTPase activities of human EF-G1mt/G2mt and *E. coli* EF-G were measured as described (34). Briefly, assays were performed in 25 μl of GTPase assay buffer (20 mM HEPES-KOH [pH 7.5], 100 mM KCl, 4.5 mM Mg(OAc)_2_, 1 mM DTT, 2 mM spermidine, 0.05 mM spermine) containing 5 pmol (final concentration: 0.2 μM) of 55S/70S ribosomes and 1 μg (final concentration 0.5 μM) of EF-G1/G2mt. Reactions were started by adding 0.5 μl of 7.5 mM [γ-^32^P]GTP (50–100 cpm/pmol). After a 10 min incubation at 30°C, the reactions were terminated by adding 100 μl of 0.1 N H_2_SO_4_–1.5 mM NaH_2_PO_4_ and 25 μl of 5% sodium molybdate. Phosphomolybdate complexes were extracted with 250 μl *n-*butanol. Aliquots (250 μl) of the butanol layer were counted by a scintillation counter. Under these conditions, the GTPase activities are detected in a linear range over time, and depend on the concentration of the factor.

## RESULTS

### Synthesis of mtDNA-encoded proteins by the reconstituted mammalian mitochondrial translation system

**Figure 1.**
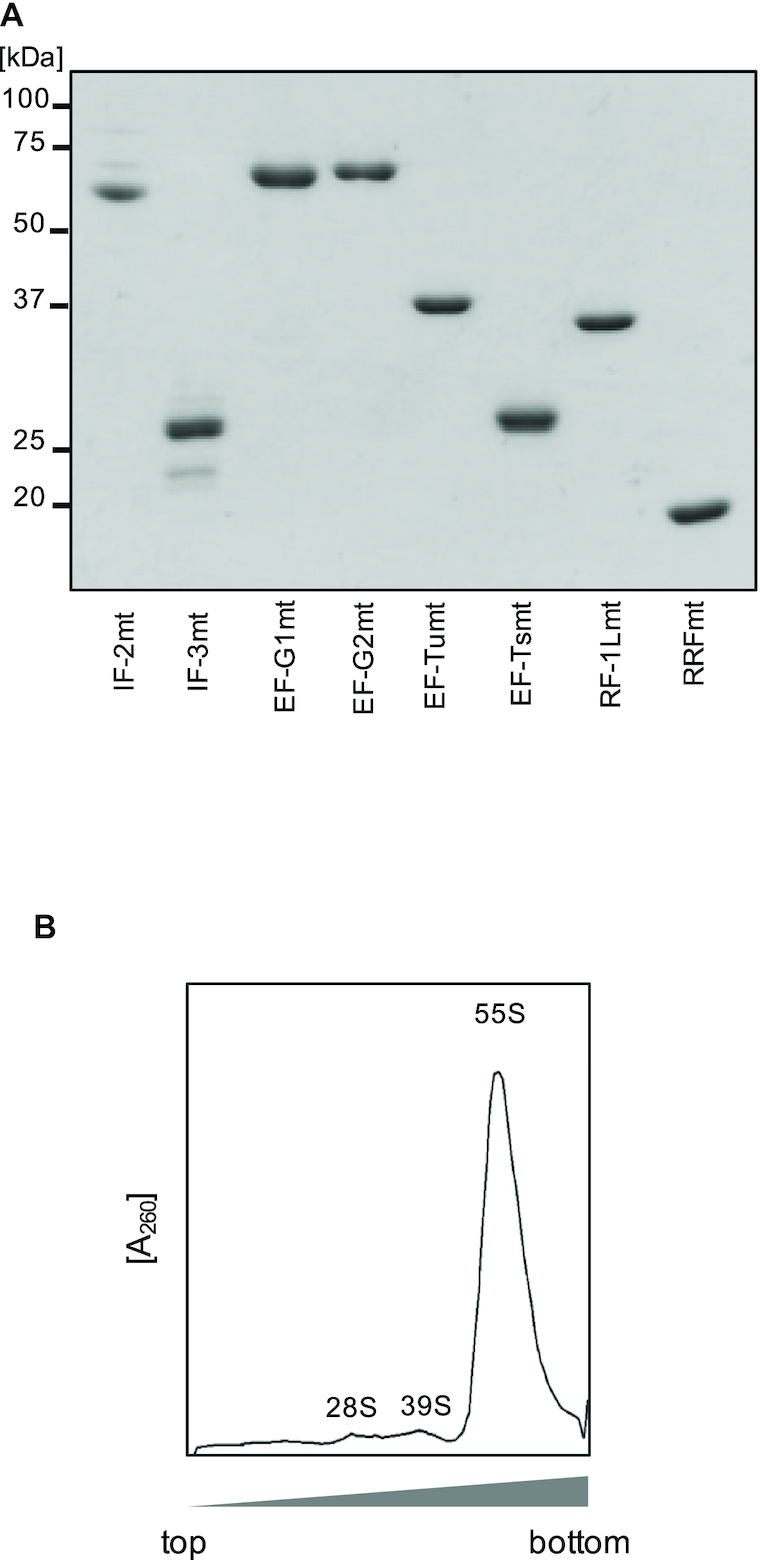
Components used in the reconstituted mammalian mitochondrial translation system. (**A**) Translation factors. Initiation factors (IF-2mt and IF-3mt), elongation factors (EF-Tumt, EF-Tsmt and EF-G1mt) and termination and recycling factors (RF-1Lmt, EF-G2mt and RRFmt), 2 μg each, were resolved by 12% SDS-PAGE and stained with Coomassie Brilliant Blue (CBB). (**B**) Ribosomes. 55S ribosomes were separated on 6–38% (w/v) sucrose gradients while monitoring the absorbance at 260 nm.

**Figure 2.**
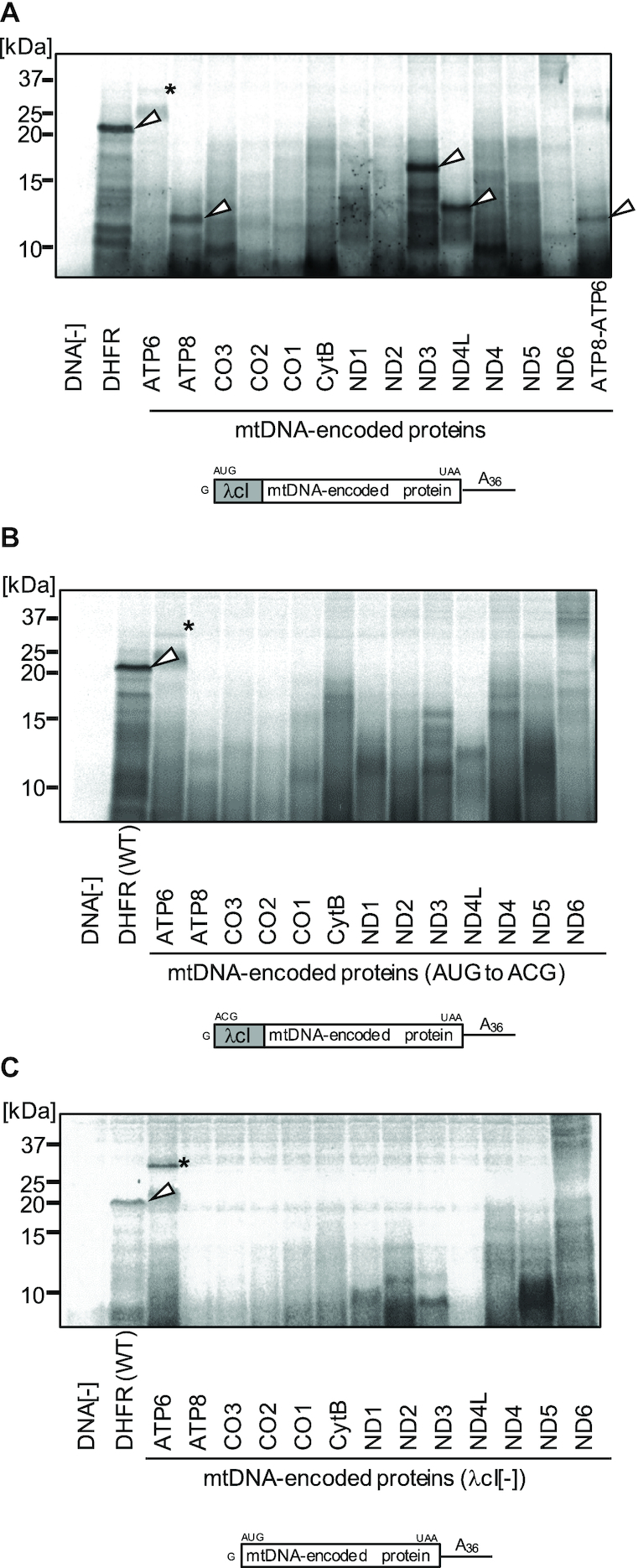
Synthesis of mtDNA-encoded proteins by the reconstituted mammalian mitochondrial translation system. (A–C) Translation reactions (coupled transcription, aminoacylation, and translation reactions) were performed using the indicated template DNA and *E. coli* tRNA mix in the presence of [^35^S] methionine. After the 120 min reaction, the samples were treated with RNase A and fractionated by 15% SDS-PAGE. The schematic of the mRNA produced from the template DNA during the reaction is shown below the panel. (**A**) Reactions with the standard template DNA; (**B**) reactions with the control template DNA, in which the initiation AUG codon in the standard template DNA is mutated to ACG and (**C**) reactions with the control template DNA, in which the λcI sequence in the standard template DNA is deleted. Full gel images are shown in Supplementary Figure S2. Another set of experiment corresponding to (C) is shown in Supplementary Figure S2C. White arrowheads indicate the relevant translation products, and asterisks denote unknown translation products. DHFR, *E. coli* dihydrofolate reductase; ND1–6 and ND4L, NADH dehydrogenase subunits; ATP6 and ATP8, ATP synthase subunits; CO1–3, cytochrome *c* oxidase subunits; CytB, cytochrome b. Molecular weights of DHFR and the mtDNA-encoded proteins, without the λcI sequence (kDa): DHFR(18), ATP6(24.8), ATP8(8), CO3(30), CO2(25.6), CO1(57), CytB(42.7), ND1(36), ND2(39), ND3(13), ND4L(11), ND4(52), ND5(67), ND6(18).

We reconstituted the mitochondrial translation system utilizing purified recombinant mitochondrial translation factors (IF-2mt, IF-3mt, EF-G1mt, EF-G2mt, EF-Tumt, EF-Tsmt, RF-1Lmt and RRFmt; Figure 1A), and the isolated 55S ribosomes from pig liver mitochondria (Figure 1B). We initially attempted to synthesize the model protein DHFR, as well as 13 mtDNA-encoded proteins (Figure 2) (full gel images are also shown in Supplementary Figure S2). We designed the DNA templates for the leaderless mRNAs of the respective proteins, in which each protein is encoded following a fragment of leaderless cI mRNA from bacteriophage lambda (Figure 2A, top; Supplementary Table S1). Using these DNA templates, translation reactions were performed by coupling the transcription and aminoacylation reactions. Here, we used the *E. coli* tRNA mixture, which can be aminoacylated by the purified *E. coli* aminoacyl tRNA synthetases added to the translation reaction system, since it is currently difficult to obtain sufficient amounts of mitochondrial tRNAs. We note that the DNA templates for mtDNA-encoded proteins utilize the DNA sequence from human mtDNA, but the codons of the mitochondrial non-universal genetic code were mutated to those of the universal code: AUA(methionine) and UGA(tryptophan) codons were changed to AUG and UGG, respectively. The model protein DHFR and three mtDNA-encoded proteins (ATP8, ND3 and ND4L) were successfully synthesized (Figure 2A), and we confirmed that these proteins were synthesized in the initiation codon-dependent manner (Figure 2B). We note, however, that species indicated in Figure 2A for ATP8, ND3 and ND4L are still present in Figure 2B, albeit at a lower intensity (see also Supplementary Figure S2E). Thus, we cannot exclude the possibility that the observed products are products of alternative translation initiation.

A sequence of cI mRNA from bacteriophage lambda was necessary for the efficient synthesis of these proteins (Figure 2C). Presumably, the 5′ proximal AUG initiation codon in the mRNA would be well exposed in the context of the lambda cI mRNA sequence. All 13 mtDNA-encoded proteins are subunits of the respiratory chain complex, and possess multiple hydrophobic trans-membrane domains. Among them, three proteins with the lowest molecular weights (ATP8, 8 kDa; ND3, 13 kDa; ND4L,11 kDa), with fewer than three trans-membrane domains, were successfully synthesized. To synthesize the rest of the mtDNA-encoded proteins, it might be necessary to supply the co-translational membrane insertion machineries in the translation system. Mitochondrial protein synthesis reportedly relies on the presence of, and on the co-assembly with, the membrane-bound nuclear-derived subunits (47). Translational activators to expose the 5′ proximal AUG initiation codon in the mRNA might be also necessary. Transcript-specific translational activators are required in the yeast mitochondrial translation system (48), and microRNA is involved in the translation activation of the mtDNA-encoded mRNAs of mammalian mitochondria (49).

**Figure 3.**
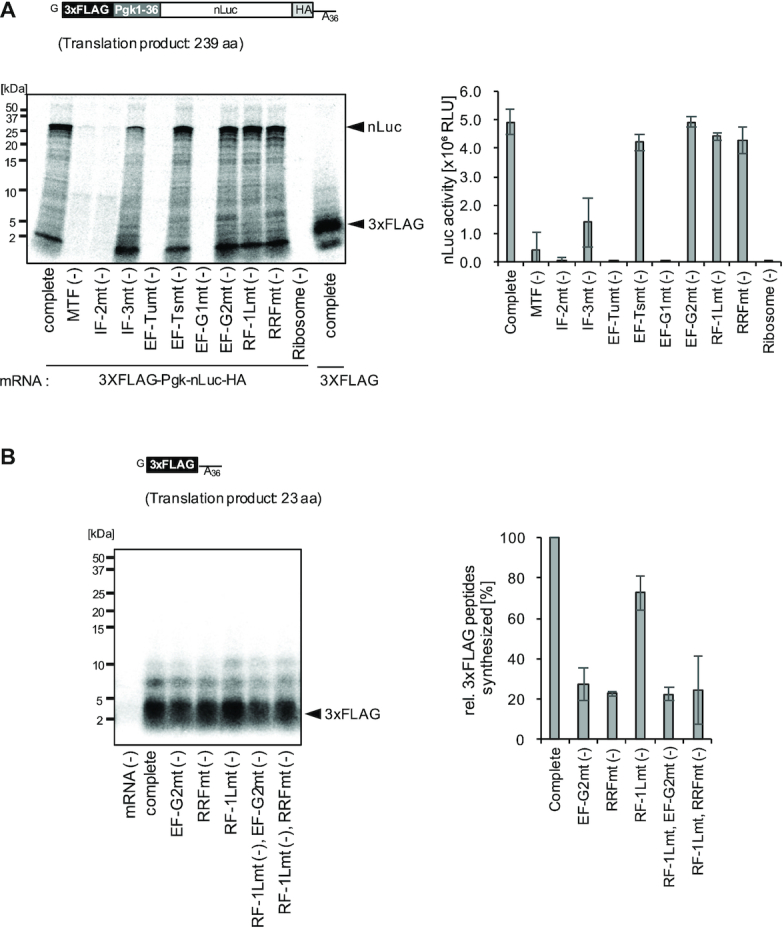
Dependence of the reconstituted mammalian mitochondrial translation system on each component. (**A**) nLuc synthesis: The schematic of the mRNA used in the reaction (upper). Translation reactions were performed using the mRNA and [^35^S]methionine-labeled yeast aminoacyl-tRNA mix, with each factor subtracted from the reaction mixture. After the 120 min reaction, either the samples were treated with RNase A and resolved by Tricine SDS-PAGE (lower left), or aliquots were subjected to the nLuc assay (lower right). The amount of nLuc synthesized in ‘Complete’ corresponds to approximately 0.026 nM. Error bars represent the standard deviation from three independent experiments. In the rightmost lane of the gel, translation product of 3XFLAG mRNA in (B) is also shown, to compare with 2–5 kDa non-specific products of nLuc mRNA (see also Supplementary Figure S4). (**B**) 3xFLAG peptide synthesis: The schematic of the mRNA used in the reaction (upper). Translation reactions were performed as in (A). After the 120 min translation reaction, the samples were treated with RNase A and resolved by Tricine SDS-PAGE (lower left). The yields of the synthesized 3xFLAG peptides were assessed by the incorporation of [^35^S]methionine into the peptide, and the amounts of the synthesized peptides at 120 min were plotted (lower right). The amount of 3xFLAG peptide synthesized in ‘Complete’ corresponds to ∼0.039 nM. Error bars represent the standard deviation from three independent experiments.

For simplicity, the mRNA constructs throughout this study were unified to have leaderless features and polyadenylation, although each mitochondrial mRNA has distinct features *in vivo* (2). In the translation of nLuciferase mRNA (used in Figures 3 and 5), addition of a few nucleotides upstream of the 5′ proximal AUG represses the translation to ∼20%, consistent with the previous report (28), and removal of polyadenylation shows minimal effect (data not shown).

### Dependence of the reconstituted mitochondrial translation system on each component

We analyzed the dependence of the reconstituted translation system on each component (Figure 3). From here on, we applied the translation system that is independent of the transcription and the aminoacylation reactions. In other words, the translation reaction was performed using the mRNA and the pre-charged yeast aminoacyl-tRNA mixture. We prepared a leaderless mRNA encoding nanoLuciferase(nLuc) flanked by the leader sequence, composed of 3xFLAG and the N-terminal 36 amino acids of yeast Pgk1 (phosphoglycerate kinase 1) (Pgk1-36), and the HA-tag (Figure 3A, upper). The nLuc synthesis reaction included this mRNA with each factor subtracted from the reaction mixture, and its activities were analyzed within a linear range (Figure 3A, lower).

The omission of either MTF or IF-2mt severely inhibited the nLuc synthesis. Although the degree of the initiator Met-tRNA formylation in the reaction is currently unknown, these results indicated that the translation is initiated with formyl-Met-tRNA in our system. The omission of IF-3mt also strongly inhibited the nLuc synthesis, in agreement with the previous report that IF-3mt stimulates the formation of initiation complexes using 55S mitoribosomes and leaderless mRNA (28). Partial inhibition of the nLuc synthesis may indicate that the translation initiation is a mixture of mode that requires and that do not require IF-3mt.

The omission of elongation factor EF-Tumt or EF-G1mt resulted in essentially no translation, while that of EF-Tsmt had minimal effects. We previously observed that EF-Tsmt promotes poly(Phe) synthesis when a limiting amount of EF-Tumt is present in the reaction (50). The effect of EF-Tsmt was probably not detected due to the presence of excess EF-Tumt.

The omission of peptide release factor RF-1Lmt did not suppress the nLuc synthesis. As we previously confirmed the stop codon-dependent peptide release activity of RF-1Lmt on mitoribosomes (40), we examined whether the synthesized nLuc is indeed released by RF-1Lmt. This analysis revealed that the synthesized nLuc is released from the ribosome even in the absence of RF-1Lmt (Supplementary Figure S3A), and thus could be a reason why RF-1Lmt minimally affected nLuc synthesis. At present, we cannot explain the precise mechanism for this RF-1Lmt-independent peptide release at the stop codon. However, as we discuss later, such unusual peptide release could occur in our system, especially when the ribosomal A-site is empty for a long time.

The omission of recycling factors (EF-G2mt, RRFmt) did not suppress the nLuc synthesis. Since these factors are capable of recycling mitoribosomes (35), one possibility is that nLuc is synthesized in a single-round of translation under the conditions of the system. Thus, we also analyzed the dependence of the synthesis of shorter peptides on these factors (Figure 3B), because oligopeptides are reportedly synthesized by multi-round translation in the reconstituted *E. coli* and yeast translation systems (40,51). We prepared the leaderless mRNA encoding 3xFLAG peptides (Figure 3B, upper). After the translation reaction was performed using the [^35^S]methionine-labeled aminoacyl-tRNA mix, the synthesized 3xFLAG peptides were resolved by Tricine SDS-PAGE, and the yields of the synthesized 3xFLAG peptides were assessed by the incorporation of [^35^S]methionine. This analysis revealed that the peptides were synthesized in an EF-G2mt- and RRFmt-dependent manner (Figure 3B, lower, EF-G2mt(–), RRFmt(–)), indicating that the synthesis proceeded by approximately two- to three-rounds of translation, as assessed by the [^35^S]methionine incorporation into the peptides. Short mRNAs also bind to the small mitoribosomal subunits, but with much lower affinity than that of long mRNAs (52). Thus, the inefficient 3xFLAG peptide synthesis of at most three-rounds might result from a slow initiation process. We note that the synthesis of oligopeptides from mRNAs shorter than 3xFLAG was not detectable (unpublished results). EF-G2mt and RRFmt dissociate ribosomes only after the peptidyl-tRNA is hydrolyzed by RF-1Lmt (40). Accordingly, multi-round synthesis of the 3xFLAG peptide should be suppressed if the peptide release by RF-1Lmt is inhibited. However, we found that the omission of RF-1Lmt did not inhibit the multi-round synthesis of the 3xFLAG peptide (Figure 3B, RF-1Lmt(–)), indicating that 3xFLAG peptides were released in an RF-1Lmt-independent manner. We examined whether the synthesized 3xFLAG peptides are released by RF-1Lmt, and found that they were indeed released from the ribosome even in the absence of RF-1Lmt (Supplementary Figure S3B), which is compatible with the RF-1Lmt-independent release of nLuc from mitoribosomes described above (Supplementary Figure S3A).

### Fusidic acid resistance of the reconstituted mitochondrial translation system

So far, the antibiotic susceptibility of the mitochondrial protein synthesis system *in vitro* has been investigated using the poly(Phe) synthesis system, a reconstituted translation elongation process (53–55). By applying the developed mitochondrial translation system, we attempted to test the antibiotics that affect the translation process other than elongation step.

**Figure 4.**
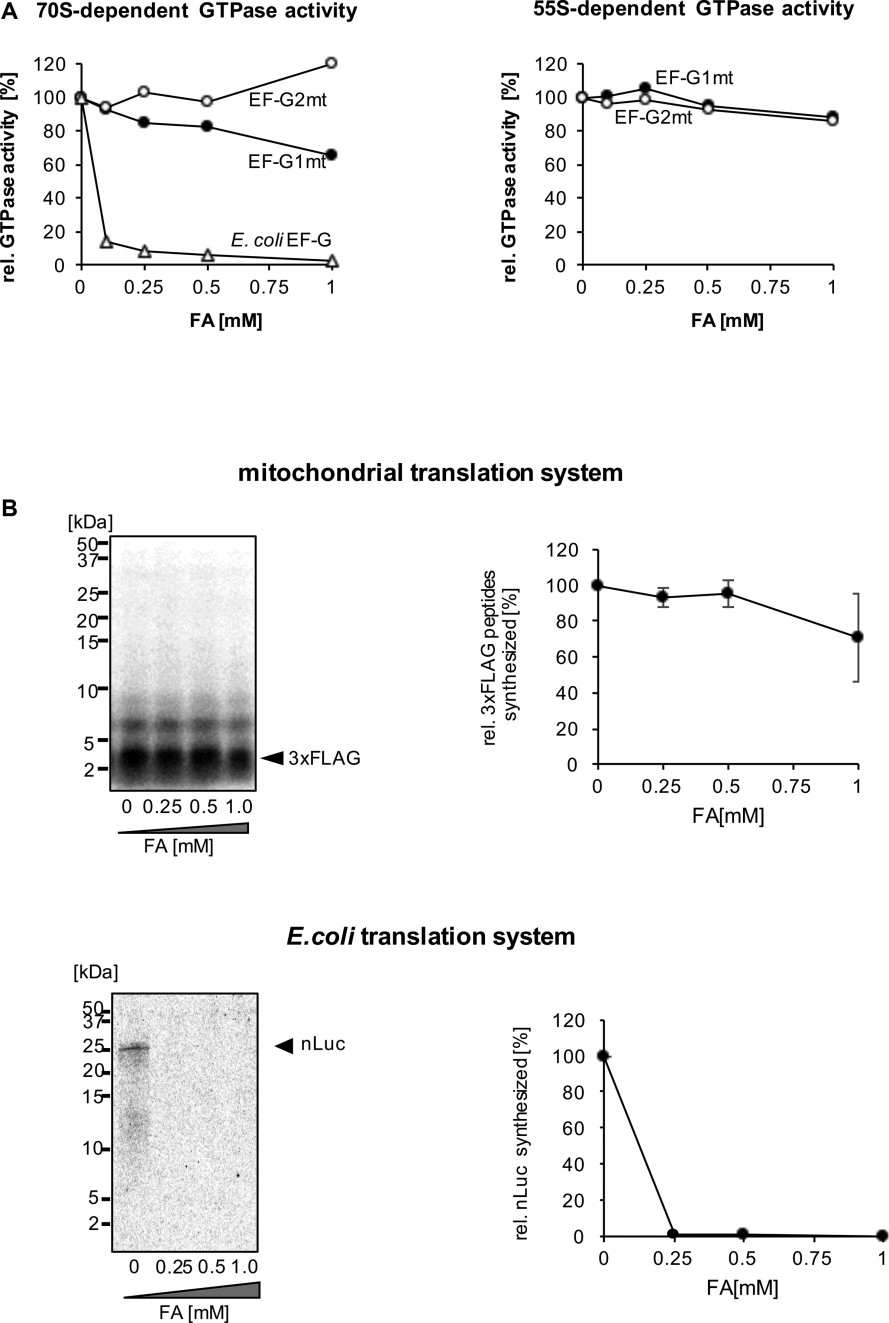
Fusidic acid (FA) resistance of the reconstituted mammalian mitochondrial translation system. (**A**) Effect of FA on ribosome-dependent GTPase activity. *E. coli* EF-G/EF-G1mt/EF-G2mt (final concentration 0.5 μM) were incubated with ribosomes (final concentration 0.2 μM) and [γ-^32^P]GTP in the presence of the indicated amounts of FA, and the [^32^P]Pi release was measured. Left, assay with *E. coli* 70S ribosomes; right, assay with mitochondrial 55S ribosomes. The amounts of Pi released at 0 mM FA correspond to 1,400 pmol (*E. coli* EF-G), 650 pmol (EF-G1mt), and 170 pmol (EF-G2mt) on 70S ribosomes, and 4,300 pmol (EF-G1mt) and 1,600 pmol (EF-G2mt) on 55S ribosomes. Note that the GTPase activity of *E. coli* EF-G is not detectable on 55S ribosomes. (**B**) Effect of FA on 3xFLAG peptide synthesis (upper). 3xFLAG peptides were synthesized as in Figure 3B, in the presence of the indicated amounts of FA. After the 120 min translation reaction, the samples were treated with RNase A and resolved by Tricine SDS-PAGE (upper left), and the amounts of the synthesized 3xFLAG peptides were plotted (upper right). Error bars represent the standard deviation from three independent experiments. Effect of FA on the nLuc synthesis with *E. coli* translation system (PURE frex ver. 2.0) was similarly analyzed for reference (lower). The standard deviation from three independent experiments do not exceed 0.3%.

One of the characteristics of EF-G homologs is their sensitivity to fusidic acid (FA). FA locks EF-G in the GTP conformation after GTP hydrolysis, and thereby prevents the factor from dissociating from the ribosome (56–58). Thus, FA inhibits multi-round translocation (59–62), as well as ribosome recycling by bacterial EF-G (63–65). The translocase EF-G1mt is reportedly resistant to FA in poly(U)-dependent poly(Phe) synthesis (53–55). An insertion in the switch1 loop of the EF-G1mt G domain may cause the decreased FA binding to EF-G1mt on the mitoribosome (66). However, the FA sensitivity of the ribosome-dependent GTPase activity by EF-G1mt has not been demonstrated. The FA susceptibility of the recycling factor EF-G2mt has scarcely been studied, and the insertion in the switch1 loop of the G domain is absent in EF-G2mt. We examined the ribosome-dependent GTPase activities of EF-G1mt and EF-G2mt, and found that FA did not affect either of them (Figure 4A). As a consequence, FA exerts minimal effects on the multi-round translation of 3xFLAG peptides, where both EF-G1mt and EF-G2mt are required (Figure 4B). The underlying mechanism for the FA resistance of EF-G1mt and EF-G2mt requires further investigation.

### Polyproline-mediated ribosome stalling in the reconstituted mitochondrial translation system

**Figure 5.**
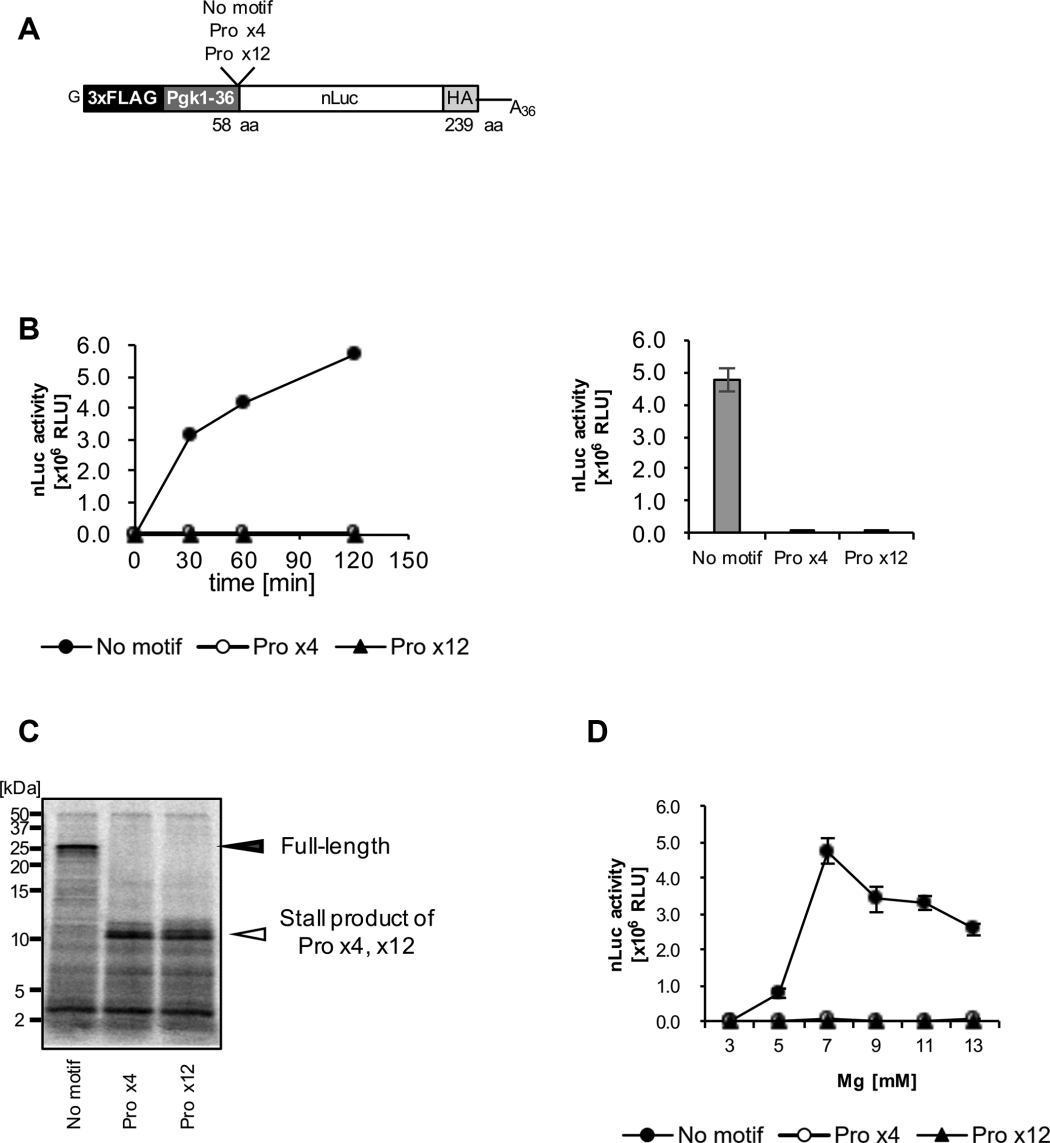
Polyproline-mediated ribosome stalling in the reconstituted mammalian mitochondrial translation system. (**A**) The schematic of the mRNA used in the reaction. A polyproline motif (Pro x4 or Pro x12) was inserted in front of the nLuc coding region. (**B**) Translation reactions were performed using the indicated mRNA and [^35^S]methionine-labeled yeast aminoacyl-tRNA mix, with 7 mM Mg^2+^. After the translation reaction was performed for the indicated time period, aliquots were subjected to the nLuc assay (left). The nLuc activities at the 120 min reaction point were plotted (right). Error bars represent the standard deviation from three independent experiments. (**C**) Translation reactions were performed as in (B). After the 120 min reaction, the samples were treated with RNase A and subjected to Tricine SDS-PAGE. Black arrowhead, full-length translation products; white arrowhead, stall products. (**D**) Translation reactions were performed as in (B), but with the indicated Mg^2+^ concentrations. The nLuc activities at 120 min were plotted. Error bars represent the standard deviation from three independent experiments.

The synthesis of proteins containing polyproline residues leads to translation arrest, which is rescued by elongation factor a/eIF5A in archaea and eukaryotes and by elongation factor P (EF-P) in bacteria (67–70). No more than three consecutive proline residues are found in mtDNA-encoded proteins, and no mitochondrial homologs of EF-P or a/eIF5A have been identified in the genome sequence. We wondered if mitochondrial ribosomes synthesizing a polyproline containing protein become stalled. We prepared mRNAs in which polyproline-motifs, either Pro x4 or Pro x12, were inserted in the middle of the coding sequences, downstream of 3xFLAG and the Pgk1–36 sequence, and upstream of the nanoLuciferase (Figure 5A), and analyzed the synthesis of nLuc from these mRNAs. We found that the Pro x4 and Pro x12 sequences both severely repressed the translation of nLuc (Figure 5B). Moreover, [^35^S]methionine-labeled truncated proteins from polyproline-containing mRNAs were observed by a Tricine SDS-PAGE analysis (Figure 5C). These results indicate that mitochondrial ribosomes indeed become stalled on polyproline-motifs.

We previously observed that a polyproline-motif caused translation repression exclusively under conditions with low Mg^2+^ and polyamine concentrations in the reconstituted yeast translation system, and increasing the Mg^2+^ concentration in the reaction alleviated the polyproline-mediated ribosome arrest (51). Therefore, we examined Mg^2+^ concentrations for the translation of polyproline-containing mRNAs in the reconstituted mitochondrial translation system. The results revealed that the polyproline-mediated stalling of the mitochondrial ribosome is not alleviated by magnesium concentrations up to 13 mM (Figure 5D), indicating a unique mechanism of polyproline-mediated translation arrest in the reconstituted mitochondrial translation system.

## DISCUSSION

### Mechanism of translation initiation of leaderless mRNA in mammalian mitochondria

In the present study, we have reconstituted a mammalian mitochondrial *in vitro* translation system capable of synthesizing long polypeptides. Leaderless mRNA, in this case nanoLuciferase, is faithfully initiated without the need for any auxiliary factors other than IF-2mt and IF-3mt. For 3XFLAG sequence added to the nLuciferase constructs, we designed it to have the low GC content and to avoid the AUG codons, including the out-frame AUG codon, as much as possible. The additional translational activators are probably not required, as long as the 5′ proximal AUG initiation codon in the mRNA is well exposed and there are no other elements in the ORF that interfere with translation.

mS39, the mitochondrial ribosome-specific pentatricopeptide repeat (PPR) protein, is located at the mRNA entrance of the ribosomal small subunit, and has been proposed to promote mRNA binding through interactions with the U-rich sequence downstream from the seventh codon of mitochondrial mRNAs (19,26). Indeed, mS39 disruption results in decreased mitochondrial translation activity (71), although this partly resulted from defects in mitochondrial ribosome assembly (72). Bacterial 70S ribosomes lack the structural homolog of mS39, but the ribosomal protein S1 located at the mRNA entrance functions in recruiting mRNA by interacting with the U-rich sequence upstream of the SD-sequence in canonical mRNA (73,74). It is noteworthy, however, that S1 is not required in the initiation of leaderless mRNA (75,76). Approximately 30–50% of the 55S ribosomes in our preparation are bound with mS39 (Supplementary Figure S5), and mS39 may not be involved in the translation initiation of, at least, the nLuc leaderless mRNA. A leucine-rich PPR motif-containing protein and an SRA stem-loop-interacting RNA-binding protein complex (LRPPRC-SLIRP complex) bind to the mitochondrial mRNA coding region to expose the initiation codon at the 5′ end of the mRNA (77), and may further recruit the mRNA to the mitoribosome via interactions with mS39 (78). The LRPPRC-SLIRP•mS39•mRNA interaction may be specifically required for the translation initiation of highly structured mitochondrial mRNAs. Further research is required to clarify the function of mS39.

### Mechanism of polyproline-mediated translation arrest in the reconstituted mitochondrial translation system

Using an *in vitro* yeast reconstituted translation system, we previously demonstrated that polyproline arrests translation in a manner similar to ‘intrinsic ribosome destabilization (IRD)’ (51). IRD is a recently discovered phenomenon in bacteria, where consecutive and proline-intermitted acidic amino acids destabilize the 70S ribosome from within the peptide tunnel and abort translation (79). In a yeast reconstituted translation system, under conditions with low Mg^2+^, polyproline destabilizes both the peptidylpolyproline–tRNA itself and ribosome, and inhibits the peptidyl-transfer reaction. However, under conditions with high Mg^2+^, where ribosomes are stable, the disorder of the peptidylpolyproline-tRNA is resolved, consequently alleviating polyproline-mediated ribosome stalling (51).

In sharp contrast to such observations in the yeast translation system, the mitochondrial translation system revealed that polyproline arrests translation, even under conditions with high Mg^2+^ (Figure 5D). Moreover, we found that the peptidylpolyproline-tRNAs on the stalled 55S ribosomes are partially hydrolyzed, and nascent peptides are released (Supplementary Figure S6). As we observed the RF-1Lmt-independent peptide release at a stop codon (Supplementary Figure S3), such premature peptide release could also happen in our system, when the ribosomal A-site is empty for a long time. The factor responsible for the peptide release has yet to be identified. Here, we would like to note our observations in the *in vitro* yeast reconstituted translation system: (i) Polyproline-mediated ribosome stalling is not accompanied by peptide release, and the peptidylpolyproline-tRNA on the stalled 80S ribosomes is scarcely hydrolyzed (51). (ii) Ribosome stalling at CGA codon repeats is associated with the peptide release, i.e. premature termination (51). CGA codon repeats in the translated mRNA are known to cause ribosome stalling, due to the decoding-incompatible conformations of the mRNA in the A-site (80). Considering these observations, it is possible that polyproline-mediated translation arrest by mitoribosomes is a combined result of an impaired peptidyl-transfer reaction due to the disorder of peptidylpolyproline-tRNA, and impaired decoding in the A-site due to, for example, an unusual mRNA structure. For instance, as the Mg^2+^ concentration increases in the reaction, the disorder of the peptidylpolyproline-tRNA might be partially resolved, but premature termination occurs due to the inhibited decoding. Structural analysis of the polyproline-stalled mitoribosome nascent chain complex will clarify the underlying mechanism and provide insights for the peptidyl-transfer reaction by mitoribosomes, and eventually the polypeptide exit tunnel (PET) of the mitoribosome will be visualized. Future studies using mitochondrial tRNAs, including mt-tRNA^Ser^(AGY), in this mitochondrial translation system will be important for a more precise understanding of the peptide transfer reaction, as well as the peptide release reaction on the mitoribosomes; unique mechanisms of polyproline-mediated ribosome stalling and of the protein release observed in this study might be due to the system's use of heterologous yeast tRNAs. In combination with the translational activators, assembly factors, and the co-translational membrane insertion machineries, such as the proteoliposomes where mitoribosome membrane receptors are integrated, the developed mitochondrial translation system will lead the way to understand the translation mechanism in mammalian mitochondria.

## Supplementary Material

gkaa1165_Supplemental_File
