# Supplementary material for: Reconstitution of mammalian mitochondrial translation system capable of correct initiation and long polypeptide synthesis from leaderless mRNA
Source: Nucleic Acids Res. 2020 Dec 9;49(1):371–82. doi: 10.1093/nar/gkaa1165 (PMC7797035; doi:10.1093/nar/gkaa1165)
Supplement: gkaa1165_Supplemental_File [file gkaa1165_supplemental_file.pdf]

Supplementary Table 1. Sequences of template DNAs for the synthesis of DHFR and mtDNA-encoded proteins

, T7 promoter; _____, a fragment of leaderless cI mRNA from bacteriophage lambda; ☐, mutation from mitochondrial non-universal genetic code to universal code; ____, polyA36.

DHFR
GGGCCTAATACGACTCACTATAGATGAGCACAAAAAAGAAACCATTA**ATG**ATCAGTCTGATTGCG<br>GCGTTAGCGGTAGATCGCGTTATCGGCATGGAAAACGCCATGCCGTGGAACCTGCCTGCCGAT<br>CTCGCCTGGTTTAAACGCAACACCTTAAATAAACCCGTGATTATGGGCCGCCATACCTGGGAAT<br>CAATCGGTCGTCCGTTGCCAGGACGCAAAAATATTATCCTCAGCAGTCAACCGGGTACGGACG<br>ATCGCGTAACGTGGGTGAAGTCGGTGGATGAAGCCATCGCGGCGTGTGGTGACGTACCAGAA<br>ATCATGGTGATTGGCGGCGGTCGCGTTTATGAACAGTTCTTGCCAAAAGCGCAAAAACTGTATC<br>TGACGCATATCGACGCAGAAGTGGAAGGCGACACCCATTTCCCGGATTACGAGCCGGATGACT<br>GGGAATCGGTATTCAGCGAATTCCACGATGCTGATGCGCAGAACTCTCACAGCTATTGCTTTGA<br>GATTCTGGAGCGGCGGTAAAAAAAAAAAAAAAAAAAAAAAAAAAAAAAAAAAAAAAAA
ATP6
GGGCCTAATACGACTCACTATAGATGAGCACAAAAAAGAAACCATTA**ATG**AACGAAAATCTGTTC<br>GCTTCATTCATTGCCCCCACAATCCTAGGCCTACCCGCCGCAGTACTGATCATTCTATTTCCCCC<br>TCTATTGATCCCCACCTCCAAATATCTCATCAACAACCGACTAATCACCACCCAACAATGGCTAA<br>TCAAACTAACCTCAAAACAAATGATGACCATGCACAACACTAAAGGACGAACCTGGTCTCTTAT<br>GCTAGTATCCTTAATCATTTTTATTGCCACAACTAACCTCCTCGGACTCCTGCCTCACTCATTTAC<br>ACCAACCACCCAACTATCTATGAACCTAGCCATGGCCATCCCCTTATGGGCGGGCACAGTGATT<br>ATGGGCTTTCGCTCTAAGATTAAAAATGCCCTAGCCCACTTCTTACCACAAGGCACACCTACAC<br>CCCTTATCCCCATGCTAGTTATTATCGAAACCATCAGCCTACTCATTCAACCAATGGCCCTGGCC<br>GTACGCCTAACCGCTAACATTACTGCAGGCCACCTACTCATGCACCTAATTGGAAGCGCCACCC<br>TAGCAATGTCAACCATTAACCTTCCCTCTACACTTATCATCTTCACAATTCTAATTCTACTGACTAT<br>CCTAGAAATCGCTGTCGCCTTAATCCAAGCCTACGTTTTCACACTTCTAGTAAGCCTCTACCTGC<br>ACGACAACACATAAAAAAAAAAAAAAAAAAAAAAAAAAAAAAAAAAAAAAAAAA
ATP8
GGGCCTAATACGACTCACTATAGATGAGCACAAAAAAGAAACCATTA**ATG**CCCCAACTAAATACT<br>ACCGTATGGCCCACCATGATTACCCCCATGCTCCTTACACTATTCCTCATCACCCAACTAAAAAT<br>GTTAAACACAAACTACCACCTACCTCCCTCACCAAAGCCCATGAAAATGAAAAATTATAACAAAC<br>CCTGGGAACCAAAATGGACGAAAATCTGTTCGCTTCATTCATTGCCCCCACAATCCTAGAAAAA<br>AAAAAAAAAAAAAAAAAAAAAAAAAAAAAAA
CO3
GGGCCTAATACGACTCACTATAGATGAGCACAAAAAAGAAACCATTA**ATG**ACCCACCAATCACAT<br>GCCTATCATATGGTAAAACCCAGCCCATGGCCCCTAACAGGGGCCCTCTCAGCCCTCCTAATG<br>ACCTCCGGCCTAGCCATGTGGTTTCACTTCCACTCCATGACGCTCCTCATGCTAGGCCTACTA<br>ACCAACACACTAACCATGTACCAATGGTGGCGCGATGTAACACGAGAAAGCACATACCAAGGC

CACCACACACCACCTGTCCAAAAAGGCCTTCGATACGGGATGATCCTATTTATTACCTCAGAAG<br>TTTTTTTCTTCGCAGGATTTTTCTGGGCCTTTTACCACTCCAGCCTAGCCCCTACCCCCCAATTA<br>GGAGGGCACTGGCCCCCAACAGGCATCACCCCGCTAAATCCCCTAGAAGTCCCACTCCTAAAC<br>ACATCCGTATTACTCGCATCAGGAGTATCAATCACCTGGGCTCACCATAGTCTAATGGAAAACAA<br>CCGAAACCAAATGATTCAAGCACTGCTTATTACAATTTTACTGGGTCTCTATTTTACCCTCCTACA<br>AGCCTCAGAGTACTTCGAGTCTCCCTTCACCATTTCCGACGGCATCTACGGCTCAACATTTTTT<br>GTAGCCACAGGCTTCCACGGACTTCACGTCATTATTGGCTCAACTTTCCTCACTATCTGCTTCAT<br>CCGCCAACTAATGTTTCACTTTACATCCAAACATCACTTTGGCTTCGAAGCCGCCGCCTGGTAC<br>TGGCATTTTGTAGATGTGGTTTGGCTATTTCTGTATGTCTCCATCTATTGGTGGGGGTCTTAAAA<br>AAAAAAAAAAAAAAAAAAAAAAAAAAAAAAAAAA
CO2
GGGCCTAATACGACTCACTATAGATGAGCACAAAAAAGAAACCATTA**ATG**GCACATGCAGCGCA<br>AGTAGGTCTACAAGACGCTACTTCCCCTATCATGGAAGAGCTTATCACCTTTCATGATCACGCC<br>CTCATGATCATTTTCCTTATCTGCTTCCTAGTCCTGTATGCCCTTTTCCTAACACTCACAACAAAA<br>CTAACTAATACTAACATCTCAGACGCTCAGGAAATGGAAACCGTCTGGACTATCCTGCCCGCCA<br>TCATCCTAGTCCTCATCGCCCTCCCATCCCTACGCATCCTTTACATGACAGACGAGGTCAACGA<br>TCCCTCCCTTACCATCAAATCAATTGGCCACCAATGGTACTGGACCTACGAGTACACCGACTAC<br>GGCGGACTAATCTTCAACTCCTACATGCTTCCCCCATTATTCCTAGAACCAGGCGACCTGCGAC<br>TCCTTGACGTTGACAATCGAGTAGTACTCCCGATTGAAGCCCCCATTCGTATGATGATTACATCA<br>CAAGACGTCTTGCACTCATGGGCTGTCCCCACATTAGGCTTAAAAACAGATGCAATTCCCGGA<br>CGTCTAAACCAAACCACTTTCACCGCTACACGACCGGGGGTATACTACGGTCAATGCTCTGAAA<br>TCTGTGGAGCAAACCACAGTTTCATGCCCATCGTCCTAGAATTAATTCCCCTAAAAATCTTTGAA<br>ATGGGGCCCGTATTTACCCTATAGAAAAAAAAAAAAAAAAAAAAAAAAAAAAAAAAAAAA
CO1
GGGCCTAATACGACTCACTATAGATGAGCACAAAAAAGAAACCATTA**ATG**TTCGCCGACCGTTG<br>GCTATTCTCTACAAACCACAAAGACATTGGAACACTATACCTATTATTCGGCGCATGGGCTGGA<br>GTCCTAGGCACAGCTCTAAGCCTCCTTATTCGAGCCGAGCTGGGCCAGCCAGGCAACCTTCTA<br>GGTAACGACCACATCTACAACGTTATCGTCACAGCCCATGCATTTGTAATGATCTTCTTCATGGT<br>AATGCCCATCATGATCGGAGGCTTTGGCAACTGGCTAGTTCCCCTAATGATCGGTGCCCCCGA<br>TATGGCGTTTCCCCGCATGAACAACATGAGCTTCTGGCTCTTACCTCCCTCTCTCCTACTCCTG<br>CTCGCATCTGCTATGGTGGAGGCCGGAGCAGGAACAGGTTGGACAGTCTACCCTCCCTTAGC<br>AGGGAACTACTCCCACCCTGGAGCCTCCGTAGACCTAACCATCTTCTCCTTACACCTAGCAGGT<br>GTCTCCTCTATCTTAGGGGCCATCAATTTCATCACAACAATTATCAATATGAAACCCCCTGCCAT<br>GACCCAATACCAAACGCCCCTCTTCGTCTGGTCCGTCCTAATCACAGCAGTCCTACTTCTCCTA<br>TCTCTCCCAGTCCTAGCTGCTGGCATCACTATGCTACTAACAGACCGCAACCTCAACACCACCT<br>TCTTCGACCCCGCCGGAGGAGGAGACCCCATTCTATACCAACACCTATTCTGGTTTTTCGGTCA<br>CCCTGAAGTTTATATTCTTATCCTACCAGGCTTCGGAATGATCTCCCATATTGTAACTTACTACTC

CGGAAAAAAAGAACCATTTGGATACATGGGTATGGTCTGGGCTATGATGTCAATTGGCTTCCTA<br>GGGTTTATCGTGTGGGCACACCATATGTTTACAGTAGGAATGGACGTAGACACACGAGCATATT<br>TCACCTCCGCTACCATGATCATCGCTATCCCCACCGGCGTCAAAGTATTTAGCTGGCTCGCCAC<br>ACTCCACGGAAGCAATATGAAATGGTCTGCTGCAGTGCTCTGGGCCCTAGGATTCATCTTTCTT<br>TTCACCGTAGGTGGCCTGACTGGCATTGTATTAGCAAACTCATCACTAGACATCGTACTACACG<br>ACACGTACTACGTTGTAGCCCACTTCCACTATGTCCTATCAATGGGAGCTGTATTTGCCATCATG<br>GGAGGCTTCATTCACTGGTTTCCCCTATTCTCAGGCTACACCCTAGACCAAACCTACGCCAAAA<br>TCCATTTCACTATCATGTTCATCGGCGTAAATCTAACTTTCTTCCCACAACACTTTCTCGGCCTAT<br>CCGGAATGCCCCGACGTTACTCGGACTACCCCGATGCATACACCACATGGAACATCCTATCATC<br>TGTAGGCTCATTCATTTCTCTAACAGCAGTAATGTTAATGATTTTCATGATTTGGGAAGCCTTCG<br>CTTCGAAGCGAAAAGTCCTAATGGTAGAAGAACCCTCCATGAACCTGGAGTGGCTATATGGAT<br>GCCCCCCACCCTACCACACATTCGAAGAACCCGTATACATGAAATCTAGAAAAAAAAAAAAAA<br>AAAAAAAAAAAAAAAAAAAAAA
CYTB
GGGCCTAATACGACTCACTATAGATGAGCACAAAAAAGAAACCATTA**ATG**ACCCCAATGCGCAA<br>AACTAACCCCCTAATGAAATTAATTAACCACTCATTCATCGACCTCCCCACCCCATCCAACATCT<br>CCGCATGGTGGAACTTCGGCTCACTCCTTGGCGCCTGCCTGATCCTCCAAATCACCACAGGA<br>CTATTCCTAGCCATGCACTACTCACCAGACGCCTCAACCGCCTTTTCATCAATCGCCCACATCA<br>CTCGAGACGTAAATTATGGCTGGATCATCCGCTACCTTCACGCCAATGGCGCCTCAATGTTCTT<br>TATCTGCCTCTTCCTACACATCGGGCGAGGCCTATATTACGGATCATTTCTCTACTCAGAAACCT<br>GGAACATCGGCATTATCCTCCTGCTTGCAACTATGGCAACAGCCTTCATGGGCTATGTCCTCCC<br>GTGGGGCCAAATGTCATTCTGGGGGGCCACAGTAATTACAAACTTACTATCCGCCATCCCATAC<br>ATTGGGACAGACCTAGTTCAATGGATCTGGGGAGGCTACTCAGTAGACAGTCCCACCCTCACA<br>CGATTCTTTACCTTTCACTTCATCTTGCCCTTCATTATTGCAGCCCTAGCAACACTCCACCTCCT<br>ATTCTTGCACGAAACGGGATCAAACAACCCCCTAGGAATCACCTCCCATTCCGATAAAATCACC<br>TTCCACCCTTACTACACAATCAAAGACGCCCTCGGCTTACTTCTCTTCCTTCTCTCCTTAATGAC<br>ATTAACACTATTCTCACCAGACCTCCTAGGCGACCCAGACAATTATACCCTAGCCAACCCCTTAA<br>ACACCCCTCCCCACATCAAGCCCGAATGGTATTTCCTATTCGCCTACACAATTCTCCGATCCGT<br>CCCTAACAAACTAGGAGGCGTCCTTGCCCTATTACTATCCATCCTCATCCTAGCAATGATCCCCA<br>TCCTCCATATGTCCAAACAACAAAGCATGATGTTTCGCCCACTAAGCCAATCACTTTATTGGCT<br>CCTAGCCGCAGACCTCCTCATTCTAACCTGGATCGGAGGACAACCAGTAAGCTACCCTTTTACC<br>ATCATTGGACAAGTAGCATCCGTACTATACTTCACAACAATCCTAATCCTAATGCCAACTATCTCC<br>CTAATTGAAAACAAAATGCTCAAATGGGCCTAAAAAAAAAAAAAAAAAAAAAAAAAAAAAAAAAA<br>AAAA
ND1
GGGCCTAATACGACTCACTATAGATGAGCACAAAAAAGAAACCATTA**ATG**CCCATGGCCAACCT<br>CCTACTCCTCATTGTACCCATTCTAATCGCAATGGCATTCCTAATGCTTACCGAACGAAAAATTCT

AGGCTATATGCAACTACGCAAAGGCCCCAACGTTGTAGGCCCCTACGGGCTACTACAACCCTT
CGCTGACGCCATGAAACTCTTCACCAAAGAGCCCCTAAAACCCGCCACATCTACCATCACCCT
CTACATCACCGCCCCGACCTTAGCTCTCACCATCGCTCTTCTACTATGGACCCCCCTCCCCATG
CCCAACCCCCTGGTCAACCTCAACCTAGGCCTCCTATTTATTCTAGCCACCTCTAGCCTAGCCG
TTTACTCAATCCTCTGGTCAGGGTGGGCATCAAACTCAAACTACGCCCTGATCGGCGCACTGC
GAGCAGTAGCCCAAACAATCTCATATGAAGTCACCCTAGCCATCATTCTACTATCAACATTACTAA
TGAGTGGCTCCTTTAACCTCTCCACCCTTATCACAACACAAGAACACCTCTGGTTACTCCTGCC
ATCATGGCCCTTGGCCATGATGTGGTTTATCTCCACACTAGCAGAGACCAACCGAACCCCCTT
CGACCTTGCCGAAGGGGAGTCCGAACTAGTCTCAGGCTTCAACATCGAATACGCCGCAGGCC
CCTTCGCCCTATTCTTCATGGCCGAATACACAAACATTATTATGATGAACACCCTCACCACTACA
ATCTTCCTAGGAACAACATATGACGCACTCTCCCCTGAACTCTACACAACATATTTTGTCACCAA
GACCCTACTTCTAACCTCCCTGTTCTTATGGATTCGAACAGCATACCCCCGATTCCGCTACGAC
CAACTCATGCACCTCCTATGGAAAAACTTCCTACCACTCACCCTAGCATTACTTATGTGGTATGT
CTCCATGCCCATTACAATCTCCAGCATTCCCCCTCAAACCTAAAAAAAAAAAAAAAAAAAAAAAA
AAAAAAAAAAAAAA

ND2

GGGCCTAATACGACTCACTATAGATGAGCACAAAAAAGAAACCATTA**ATG**AATCCCCTGGCCCA
ACCCGTCATCTACTCTACCATCTTTGCAGGCACACTCATCACAGCGCTAAGCTCGCACTGGTTT
TTTACCTGGGTAGGCCTAGAAATGAACATGCTAGCTTTTATTCCAGTTCTAACCAAAAAAATGAA
CCCTCGTTCCACAGAAGCTGCCATCAAGTATTTCCTCACGCAAGCAACCGCATCCATGATCCTT
CTAATGGCTATCCTCTTCAACAATATGCTCTCCGGACAATGGACCATGACCAATACTACCAATCA
ATACTCATCATTAATGATCATGATGGCTATGGCAATGAAACTAGGAATGGCCCCCTTTCACTTCT
GGGTCCCAGAGGTTACCCAAGGCACCCCTCTGACATCCGGCCTGCTTCTTCTCACATGGCAA
AAACTAGCCCCCATCTCAATCATGTACCAAATCTCTCCCTCACTAAACGTAAGCCTTCTCCTCAC
TCTCTCAATCTTATCCATCATGGCAGGCAGTTGGGGTGGATTAAACCAAACCCAGCTACGCAAA
ATCTTAGCATACTCCTCAATTACCCACATGGGATGGATGATGGCAGTTCTACCGTACAACCCTA
ACATGACCATTCTTAATTTAACTATTTATATTATCCTAACTACTACCGCATTCCTACTACTCAACTTA
AACTCCAGCACCACGACCCTACTACTATCTCGCACCTGGAACAAGCTAACATGGCTAACACCC
TTAATTCCATCCACCCTCCTCTCCCTAGGAGGCCTGCCCCCGCTAACCGGCTTTTTGCCCAAAT
GGGCCATTATCGAAGAATTCACAAAAAACAATAGCCTCATCATCCCCACCATCATGGCCACCAT
CACCCTCCTTAACCTCTACTTCTACCTACGCCTAATCTACTCCACCTCAATCACACTACTCCCCA
TGTCTAACAACGTAAAAATGAAATGGCAGTTTGAACATACAAAACCCACCCCATTCCTCCCCAC
ACTCATCGCCCTTACCACGCTACTCCTACCTATCTCCCCTTTTATGCTAATGATCTTATAAAAAAA
AAAAAAAAAAAAAAAAAAAAAAAAAAAAAAA

ND3

GGGCCTAATACGACTCACTATAGATGAGCACAAAAAAGAAACCATTA**ATG**AACTTCGCCTTAATT
TTAATGATCAACACCCTCCTAGCCTTACTACTAATGATTATTACATTTTGGCTACCACAACTCAAC

GGCTACATGGAAAAATCCACCCCTTACGAGTGCGGCTTCGACCCTATGTCCCCCGCCCGCGTCCCTTTCTCCATGAAATTCTTCTTAGTAGCTATTACCTTCTTATTATTTGATCTAGAAATTGCCCTCCTTTTACCCCTACCATGGGCCCTACAAACAACTAACCTGCCACTAATGGTTATGTCATCCCTCTTATTAATCATCATCCTAGCCCTAAGTCTGGCCTATGAGTGGCTACAAAAAGGATTAGACTGGACCGAATAAAAAAAAAAAAAAAAAAAAAAAAAAAAAAAAAAAAAAAAA
ND4L
GGGCCTAATACGACTCACTATAGATGAGCACAAAAAAGAAACCATTA**ATG**CCCCTCATTTACATGAATATTATGCTAGCATTTACCATCTCACTTCTAGGAATGCTAGTATATCGCTCACACCTCATGTCCTCCCTACTATGCCTAGAAGGAATGATGCTATCGCTGTTCATTATGGCTACTCTCATGACCCTCAACACCCACTCCCTCTTAGCCAATATTGTGCCTATTGCCATGCTAGTCTTTGCCGCCTGCGAAGCAGCGGTGGGCCTAGCCCTACTAGTCTCAATCTCCAACACATATGGCCTAGACTACGTACATAACCTAAACCTACTCCAATGCTAAAAAAAAAAAAAAAAAAAAAAAAAAAAAAAAAAAAAA
ND4
GGGCCTAATACGACTCACTATAGATGAGCACAAAAAAGAAACCATTA**ATG**CTAAAACTAATCGTCCCAACAATTATGTTACTACCACTGACATGGCTTTCCAAAAAACACATGATTTGGATCAACACAACCACCCACAGCCTAATTATTAGCATCATCCCTCTACTATTTTTAACCAAATCAACAACAACCTATTTAGCTGTTCCCCAACCTTTTCCTCCGACCCCCTAACAACCCCCCTCCTAATGCTAACTACCTGGCTCCTACCCCTCACAATCATGGCAAGCCAACGCCACTTATCCAGTGAACCACTATCACGAAAAAAACTCTACCTCTCTATGCTAATCTCCCTACAAATCTCCTTAATTATGACATTCACAGCCACAGAACTAATCATGTTTTATATCTTCTTCGAAACCACACTTATCCCCACCTTGGCTATCATCACCCGATGGGGCAACCAGCCAGAACGCCTGAACGCAGGCACATACTTCCTATTCTACACCCTAGTAGGCTCCCTTCCCCTACTCATCGCACTAATTTACACTCACAACACCCTAGGCTCACTAAACATTCTACTACTCACTCTCACTGCCCAAGAACTATCAAACTCCTGGGCCAACAACTTAATGTGGCTAGCTTACACAATGGCTTTTATGGTAAAGATGCCTCTTTACGGACTCCACTTATGGCTCCCTAAAGCCCATGTCGAAGCCCCCATCGCTGGGTCAATGGTACTTGCCGCAGTACTCTTAAAACTAGGCGGCTATGGTATGATGCGCCTCACACTCATTCTCAACCCCCTGACAAAACACATGGCCTACCCCTTCCTTGTACTATCCCTATGGGGCATGATTATGACAAGCTCCATCTGCCTACGACAAACAGACCTAAAATCGCTCATTGCATACTCTTCAATCAGCCACATGGCCCTCGTAGTAACAGCCATTCTCATCCAAACCCCCTGGAGCTTCACCGGCGCAGTCATTCTCATGATCGCCCACGGGCTTACATCCTCATTACTATTCTGCCTAGCAAACTCAAACTACGAACGCACTCACAGTCGCATCATGATCCTCTCTCAAGGACTTCAAACTCTACTCCCACTAATGGCTTTTTGGTGGCTTCTAGCAAGCCTCGCTAACCTCGCCTTACCCCCCACTATTAACCTACTGGGAGAACTCTCTGTGCTAGTAACCACGTTCTCCTGGTCAAATATCACTCTCCTACTTACAGGACTCAACATGCTAGTCACAGCCCTATACTCCCTCTACATGTTTACCACAACACAATGGGGCTCACTCACCCACCACATTAACAACATGAAACCCTCATTCACACGAGAAAACACCCTCATGTTCATGCACCTATCCCCCATTCTCCTCCTATCCCTCAACCCCGACATCATTACCGGGTTTTCCTCTTAAAAAAAAAAAAAAAAAAAAAAAAAAAAAAAAAAAAAAAA
ND5

GGGCCTAATACGACTCACTATAGATGAGCACAAAAAAGAAACCATTA**ATG**ACCATGCACACTACT
ATGACCACCCTAACCCTGACTTCCCTAATTCCCCCCATCCTTACCACCCTCGTTAACCCTAACA
AAAAAAACTCATACCCCCATTATGTAAAATCCATTGTCGCATCCACCTTTATTATCAGTCTCTTCC
CCACAACAATGTTCATGTGCCTAGACCAAGAAGTTATTATCTCGAACTGGCACTGGGCCACAA
CCCAAACAACCCAGCTCTCCCTAAGCTTCAAACTAGACTACTTCTCCATGATGTTCATCCCTGT
AGCATTGTTCGTTACATGGTCCATCATGGAATTCTCACTGTGGTATATGAACTCAGACCCAAAC
ATTAATCAGTTCTTCAAATATCTACTCATCTTCCTAATTACCATGCTAATCTTAGTTACCGCTAACA
ACCTATTCCAACTGTTCATCGGCTGGGAGGGCGTAGGAATTATGTCCTTCTTGCTCATCAGTTG
GTGGTACGCCCGAGCAGATGCCAACACAGCAGCCATTCAAGCAATCCTATACAACCGTATCGG
CGATATCGGTTTCATCCTCGCCTTAGCATGGTTTATCCTACACTCCAACTCATGGGACCCACAA
CAAATGGCCCTTCTAAACGCTAATCCAAGCCTCACCCCACTACTAGGCCTCCTCCTAGCAGCA
GCAGGCAAATCAGCCCAATTAGGTCTCCACCCCTGGCTCCCCTCAGCCATGGAAGGCCCCAC
CCCAGTCTCAGCCCTACTCCACTCAAGCACTATGGTTGTAGCAGGAATCTTCTTACTCATCCGC
TTCCACCCCCTAGCAGAAAATAGCCCACTAATCCAAACTCTAACACTATGCTTAGGCGCTATCAC
CACTCTGTTCGCAGCAGTCTGCGCCCTTACACAAAATGACATCAAAAAAATCGTAGCCTTCTCC
ACTTCAAGTCAACTAGGACTCATGATGGTTACAATCGGCATCAACCAACCACACCTAGCATTCC
TGCACATCTGTACCCACGCCTTCTTCAAAGCCATGCTATTTATGTGCTCCGGGTCCATCATCCA
CAACCTTAACAATGAACAAGATATTCGAAAAATGGGAGGACTACTCAAAACCATGCCTCTCACT
TCAACCTCCCTCACCATTGGCAGCCTAGCATTAGCAGGAATGCCTTTCCTCACAGGTTTCTACT
CCAAAGACCACATCATCGAAACCGCAAACATGTCATACACAAACGCCTGGGCCCTATCTATTAC
TCTCATCGCTACCTCCCTGACAAGCGCCTATAGCACTCGAATGATTCTTCTCACCCTAACAGGT
CAACCTCGCTTCCCCACCCTTACTAACATTAACGAAAATAACCCCACCCTACTAAACCCCATTAA
ACGCCTGGCAGCCGGAAGCCTATTCGCAGGATTTCTCATTACTAACAACATTTCCCCCGCATCC
CCCTTCCAAACAACAATCCCCCTCTACCTAAAACTCACAGCCCTCGCTGTCACTTTCCTAGGAC
TTCTAACAGCCCTAGACCTCAACTACCTAACCAACAAACTTAAAATGAAATCCCCACTATGCACA
TTTTATTTCTCCAACATGCTCGGATTCTACCCTAGCATCACACACCGCACAATCCCCTATCTAGG
CCTTCTTACGAGCCAAAACCTGCCCCTACTCCTCCTAGACCTAACCTGGCTAGAAAAGCTATTA
CCTAAAACAATTTCACAGCACCAAATCTCCACCTCCATCATCACCTCAACCCAAAAAGGCATGA
TTAAACTTTACTTCCTCTCTTTCTTCTTCCCACTCATCCTAACCCTACTCCTAATCACATAAAAAAA
AAAAAAAAAAAAAAAAAAAAAAAAAAAAAA

ND6

GGGCCTAATACGACTCACTATAGATGAGCACAAAAAAGAAACCATTA**ATG**ATGTATGCTTTGTTT
CTGTTGAGTGTGGGTTTAGTAATGGGGTTTGTGGGGTTTTCTTCTAAGCCTTCTCCTATTTATGG
GGGTTTAGTATTGATTGTTAGCGGTGTGGTCGGGTGTGTTATTATTCTGAATTTTGGGGGAGGTT
ATATGGGTTTAATGGTTTTTTTAATTTATTTAGGGGGAATGATGGTTGTCTTTGGATATACTACAGC
GATGGCTATTGAGGAGTATCCTGAGGCATGGGGGTCAGGGGTTGAGGTCTTGGTGAGTGTTTT
AGTGGGGTTAGCGATGGAGGTAGGATTGGTGCTGTGGGTGAAAGAGTATGATGGGGTGGTGG

TTGTGGTAAACTTTAATAGTGTAGGAAGCTGGATGATTTATGAAGGAGAGGGGTCAGGGTTGAT
TCGGGAGGATCCTATTGGTGCGGGGGCTTTGTATGATTATGGGCGTTGGTTAGTAGTAGTTACT
GGTTGGACATTGTTTGTTGGTGTATATATTGTAATTGAGATTGCTCGGGGGAATAGAAAAAAAAA
AAAAAAAAAAAAAAAAAAAAAAAAAAAA

ATP8-ATP6

GGGCCTAATACGACTCACTATAGATGAGCACAAAAAAGAAACCATTA**ATG**CCCCAACTAAATACT
ACCGTATGGCCCACCATGATTACCCCCATGCTCCTTACACTATTCCTCATCACCCAACTAAAAAT
GTTAAACACAAACTACCACCTACCTCCCTCACCAAAGCCCATGAAAATGAAAAATTATAACAAAC
CCTGGGAACCAAAATGGACGAAAATCTGTTCGCTTCATTCATTGCCCCCACAATCCTAGGCCTA
CCCGCCGCAGTACTGATCATTCTATTTCCCCCTCTATTGATCCCCACCTCCAAATATCTCATCAA
CAACCGACTAATCACCACCCAACAATGGCTAATCAAACTAACCTCAAAACAAATGATGACCATG
CACAACACTAAAGGACGAACCTGGTCTCTTATGCTAGTATCCTTAATCATTTTTATTGCCACAAC
TAACCTCCTCGGACTCCTGCCTCACTCATTTACACCAACCACCCAACTATCTATGAACCTAGCC
ATGGCCATCCCCTTATGGGCGGGCACAGTGATTATGGGCTTTCGCTCTAAGATTAAAAATGCCC
TAGCCCACTTCTTACCACAAGGCACACCTACACCCCTTATCCCCATGCTAGTTATTATCGAAACC
ATCAGCCTACTCATTCAACCAATGGCCCTGGCCGTACGCCTAACCGCTAACATTACTGCAGGCC
ACCTACTCATGCACCTAATTGGAAGCGCCACCCTAGCAATGTCAACCATTAACCTTCCCTCTAC
ACTTATCATCTTCACAATTCTAATTCTACTGACTATCCTAGAAATCGCTGTCGCCTTAATCCAAGC
CTACGTTTTCACACTTCTAGTAAGCCTCTACCTGCACGACAACACATAAAAAAAAAAAAAAAAAA
AAAAAAAAAAAAAAAAAAAA

Supplementary Table 2. Sequences of template DNAs for the nLuc and 3xFLAG synthesis reactions

(grey shading), T7 promoter; ATG, initiation Met codon; ____, 3xFLAG; ____, pgk1-36; (box), polyproline; _ _ _ _, HA; **A**x36, polyA36.

T7_Met_3XFLAG_PGK1-36 _nLuc_HA_A36
GGGCCTAATACGACTCACTATAG**ATG**GATTATAAAGATCACGACGGTGATTATAAAGATCATGATATTGATTATAAAGATGACGATGATAAAGAATTATCTTCAAAGTTGTCTGTCCAAGATTTGGACTTGAAGGACAAGCGTGTCTTCATCAGAGTTGACTTCAACGTCCCATTGGACGGTAAGAAGATCACTTCTACGGTTTTCACCTTGGAAGATTTCGTTGGTGATTGGAGACAAACTGCTGGTTACAATTTGGATCAAGTCTTGGAACAAGGTGGTGTCTCTTCTTTGTTTCAAAACTTGGGTGTTTCCGTTACCCCAATCCAAAGAATAGTTTTGTCTGGTGAAAACGGTTTGAAGATCGATATCCATGTTATCATCCCATACGAAGGTTTGTCAGGTGATCAAATGGGTCAAATCGAAAAGATCTTCAAGGTTGTTTACCCAGTTGATGATCACCACTTTAAGGTTATCTTGCACTACGGTACTTTGGTCATTGATGGTGTTACTCCAAACATGATCGATTACTTTGGTAGACCTTACGAAGGTATTGCTGTTTTCGATGGTAAGAAGATTACTGTCACTGGTACTTTGTGGAACGGTAACAAAATTATCGACGAAAGATTGATCAACCCAGACGGTTCTTTGTTGTTCAGAGTTACTATTAACGGTGTTACCGGTTGGAGATTGTGCGAAAGAATTTTGGCTTACCCATACGACGTCCCAGACTACGCGTAA**AAAAAAAAAAAAAAAAAAAAAAAAAAAAAAAAAAAA**
T7_Met_3XFLAG_PGK1-36_P4 _nLuc_HA_A36
GGGCCTAATACGACTCACTATAG**ATG**GATTATAAAGATCACGACGGTGATTATAAAGATCATGATATTGATTATAAAGATGACGATGATAAAGAATTATCTTCAAAGTTGTCTGTCCAAGATTTGGACTTGAAGGACAAGCGTGTCTTCATCAGAGTTGACTTCAACGTCCCATTGGACGGTAAGAAGATCACTTCTCCTCCCCCACCGACGGTTTTCACCTTGGAAGATTTCGTTGGTGATTGGAGACAAACTGCTGGTTACAATTTGGATCAAGTCTTGGAACAAGGTGGTGTCTCTTCTTTGTTTCAAAACTTGGGTGTTTCCGTTACCCCAATCCAAAGAATAGTTTTGTCTGGTGAAAACGGTTTGAAGATCGATATCCATGTTATCATCCCATACGAAGGTTTGTCAGGTGATCAAATGGGTCAAATCGAAAAGATCTTCAAGGTTGTTTACCCAGTTGATGATCACCACTTTAAGGTTATCTTGCACTACGGTACTTTGGTCATTGATGGTGTTACTCCAAACATGATCGATTACTTTGGTAGACCTTACGAAGGTATTGCTGTTTTCGATGGTAAGAAGATTACTGTCACTGGTACTTTGTGGAACGGTAACAAAATTATCGACGAAAGATTGATCAACCCAGACGGTTCTTTGTTGTTCAGAGTTACTATTAACGGTGTTACCGGTTGGAGATTGTGCGAAAGAATTTTGGCTTACCCATACGACGTCCCAGACTACGCGTAA**AAAAAAAAAAAAAAAAAAAAAAAAAAAAAAAAAAAA**
T7_Met_3XFLAG_PGK1-36 _P12_nLuc_HA_A36
GGGCCTAATACGACTCACTATAG**ATG**GATTATAAAGATCACGACGGTGATTATAAAGATCATGATATTGATTATAAAGATGACGATGATAAAGAATTATCTTCAAAGTTGTCTGTCCAAGATTTGGACTTGAAGGACAAGCGTGTCTTCATCAGAGTTGACTTCAACGTCCCATTGGACGGTAAGAAGATCACTTCTCCTCCCCCACCGCCTCCCCCACCGCCTCCCCCACCGACGGTTTTCACCTTGGAAGATTTCGTTGGTGATTGGAGACAAACTGCTGGTTACAATTTGGATCAAGTCTTGGAACAAGGTGGTGTCTCTTCTTTGTTTCAAAACTTGGGTGTTTCCGTTACCCCAATCCAAAGAATAGTTTTGTCTGGTGAAA

ACGGTTTGAAGATCGATATCCATGTTATCATCCCATACGAAGGTTTGTCAGGTGATCAAATGGGT<br>CAAATCGAAAAGATCTTCAAGGTTGTTTACCCAGTTGATGATCACCACTTTAAGGTTATCTTGCA<br>CTACGGTACTTTGGTCATTGATGGTGTTACTCCAAACATGATCGATTACTTTGGTAGACCTTACG<br>AAGGTATTGCTGTTTTCGATGGTAAGAAGATTACTGTCACTGGTACTTTGTGGAACGGTAACAAA<br>ATTATCGACGAAAGATTGATCAACCCAGACGGTTCTTTGTTGTTCAGAGTTACTATTAACGGTGT<br>TACCGGTTGGAGATTGTGCGAAAGAATTTTGGCTTACCCATACGACGTCCCAGACTACGCGTAA<br>**AAAAAAAAAAAAAAAAAAAAAAAAAAAAAAAAAAAA**
T7_Met_3XFLAG _A36
GGGCCTAATACGACTCACTATAG**ATG**GATTATAAAGATCACGACGGTGATTATAAAGATCATGATA<br>TTGATTATAAAGATGACGATGATAAATAA**AAAAAAAAAAAAAAAAAAAAAAAAAAAAAAAAAAA**<br>**A**

Supplementary Table 3. Summary of the expression and purification of the components used in the reconstituted mammalian mitochondrial translation system

Component	Origin	Expression vector	Histidine-Tag	Reference
IF-2mt	bovine	pET32a-BMIF2	C-terminal	This work[#1]
IF-3mt	human	pET29b-HMIF3	C-terminal	This work[#2]
EF-Tumt	bovine	pET24c-BMtu	C-terminal	Suzuki, H. (2007) *J. Biol. Chem.*
EF-Tsmt	bovine	pET24c-BMts	C-terminal	Akama, K. (2010) *Biochim. Biophys. Acta*
EF-G1mt	human	pET15b-HMEFG1	No	Tsuboi, M. (2009) *Mol. Cell*
RF1Lmt/mtRF1a	human	pET15b-HMRF1L	No	Nozaki, Y. (2008) *Genes Cells*
EF-G2mt	human	pET15b-HMEFG2	No	Tsuboi, M. (2009) *Mol. Cell*
RRFmt	human	pET15b-HMRRF	No	Tsuboi, M. (2009) *Mol. Cell*

#1: Proteins were purified according to Ma, J. (1996) *J. Biol. Chem.*

#2: Proteins were purified according to Koc, E.C. (2002) *J. Biol. Chem.*

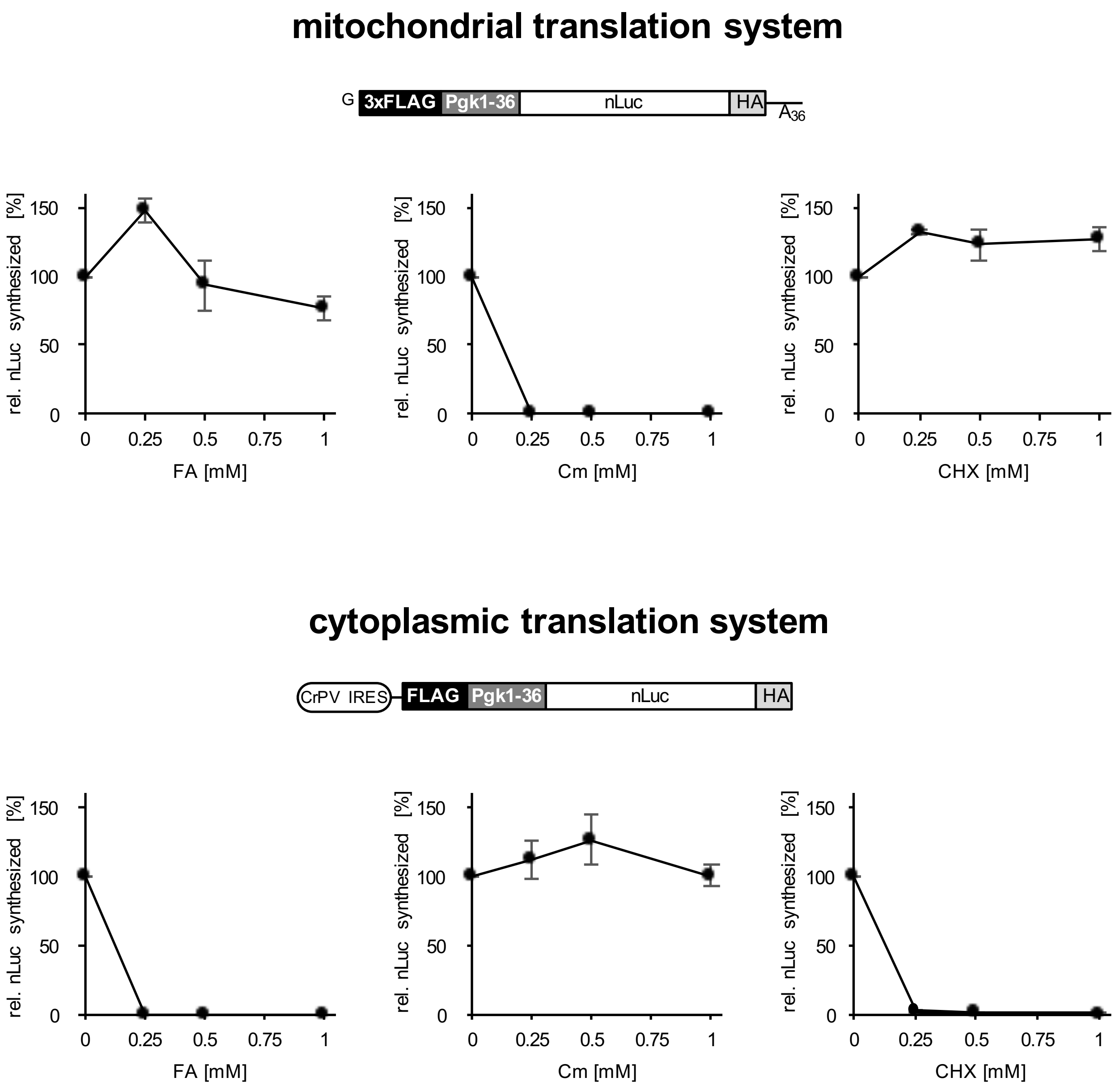

Supplementary Figure 1

Effects of fusidic acid (FA), chloramphenicol (Cm) and cycloheximide (CHX) on the reconstituted mammalian mitochondrial translation system (upper). Translation reactions were performed in the presence of indicated concentration of antibiotics for 120 min, and subjected to the nLuc assay. The stock of FA and CHX are dissolved in water, and Cm is in DMSO. The schematic of the mRNA used in the reaction is shown above the panels. Error bars represent the standard deviation from three independent experiments. Effects on the reconstituted yeast translation system were similarly analyzed for reference (lower). Details of the translation system and mRNA construct are described in (Abe T., 2020. ***J. Biochem.***).

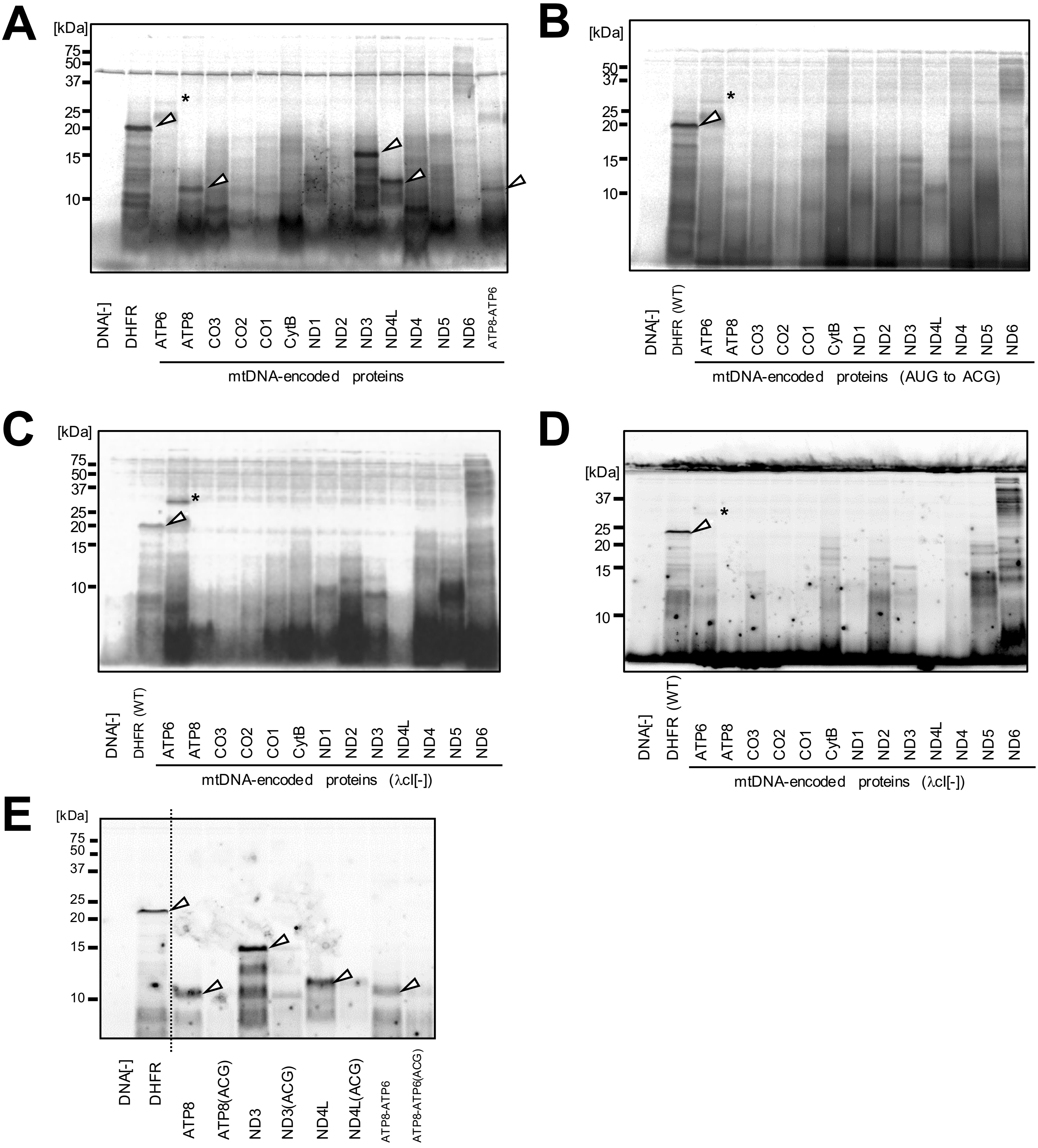

Supplementary Figure 2

(A-C) Uncropped overall views of the gel image in Figure 2A-C.
(D) Another set of experiment corresponding to Figure 2C (Supplementary Figure 2C), showing that asterisks denote unknown products.
(E) Experiments for ATP8, ND3, ND4L and ATP8-ATP6 in Figure 2A and 2B were performed in the same gel.

A

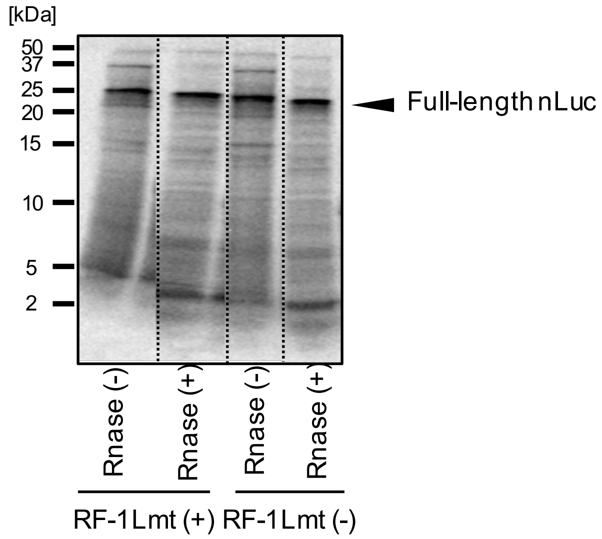

B

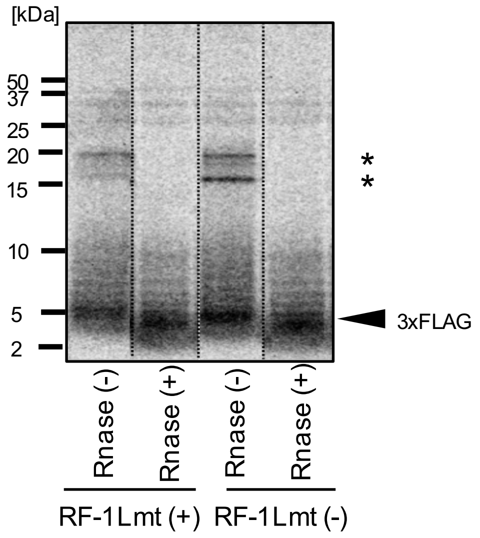

Supplementary Figure 3

nLuc (A) and 3xFLAG (B) synthesized in the absence of RF-1Lmt are released from ribosomes.

Translation reactions were performed as in Figure 5C (A) or in Figure 3B (B). After the 120 min reaction, the samples were treated with either nothing (Rnase (-)) or RNase A (Rnase(+)), and subjected to Tricine SDS-PAGE. Note that tRNA-cleaved polypeptides are significantly detected even in the RF-1Lmt(-) and Rnase(-) samples. Asterisks indicate unknown products, which presumably originated from f[^{35}S]Met-tRNA.

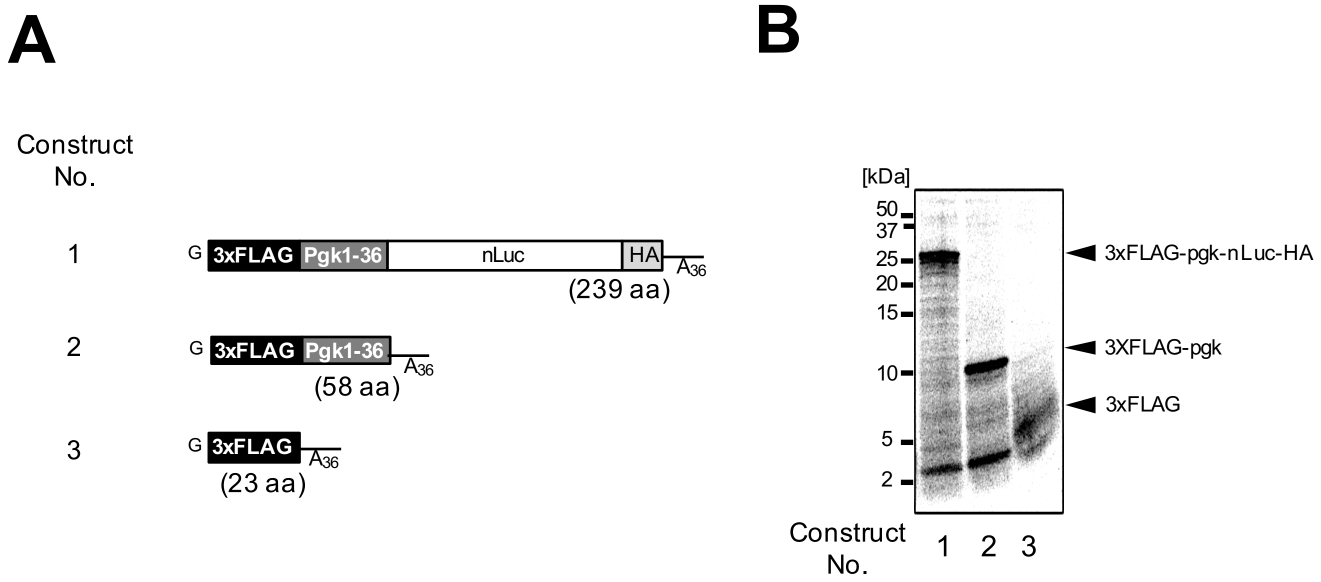

Supplementary Figure 4

Translation of mRNAs of different lengths in the reconstituted mammalian mitochondrial translation system.

(A) The schematic of the mRNA used in the reaction.

(B) Translation of the indicated mRNAs were performed as in Figure 3. The translation products corresponding to the length of the ORF are synthesized.

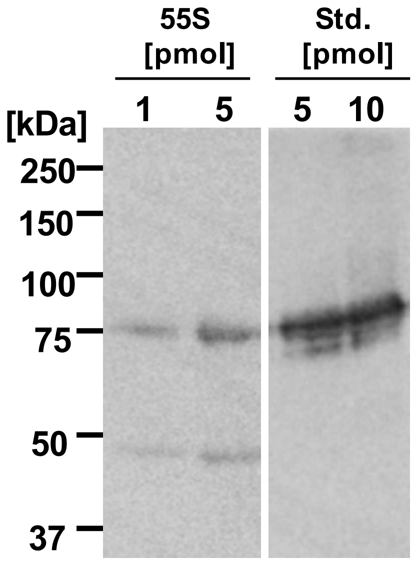

Supplementary Figure 5

Western analysis of 55S ribosomes with anti-mS39 (Invitrogen #PA5-97087). The amount of mS39 bound to the 55S ribosomes was estimated using the standard recombinant mS39, which was expressed and purified in our laboratory. mS39 is bound to approximately 30% of the 55S mitoribosomes in this lot.

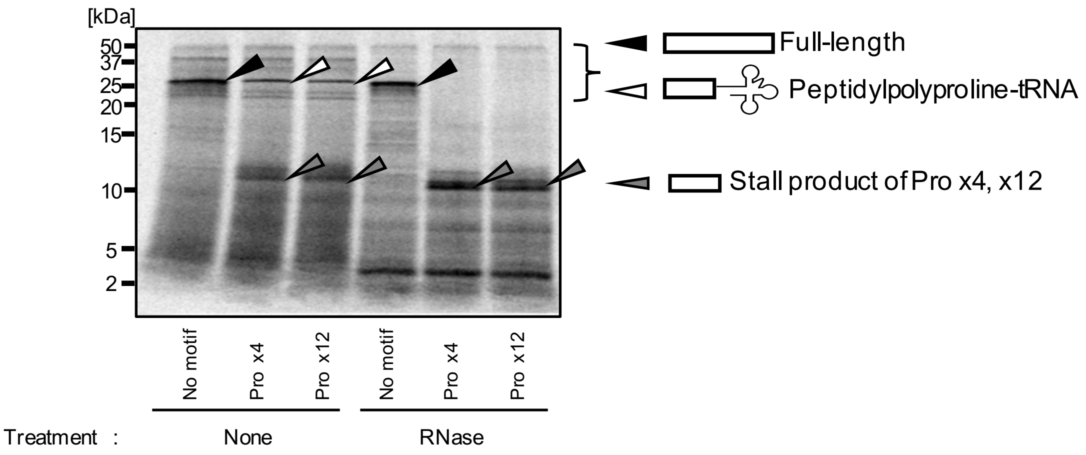

Supplementary Figure 6

Peptidylpolyproline-tRNAs on the stalled 55S ribosomes are partially hydrolyzed.

Translation reactions were performed as in Figure 5C. After the 120 min reaction, the samples were treated with either nothing (None) or RNase A (RNase), and subjected to Tricine SDS-PAGE. In the Pro x4 and Pro x12 samples, omission of the RNase treatment results in the partial disappearance of tRNA-cleaved stall products (gray arrowheads), with the concomitant appearance of peptidylproline-tRNAs (white arrowheads). In the no motif sample, tRNA-cleaved full-length products (black arrowheads) are observed regardless of the RNase treatment.
